# Atomic-Level View of the Functional Transition in Vertebrate Hemoglobins: The Case of Antarctic Fish Hbs

**DOI:** 10.1021/acs.jcim.2c00727

**Published:** 2022-08-05

**Authors:** Nicole Balasco, Antonella Paladino, Giuseppe Graziano, Marco D’Abramo, Luigi Vitagliano

**Affiliations:** † Institute of Molecular Biology and Pathology, CNR c/o Dep. Chemistry, University of Rome, Sapienza, P.le A. Moro 5, 00185 Rome, Italy; ‡ Institute of Biostructures and Bioimaging, CNR, Via Pietro Castellino 111, 80131 Naples, Italy; § Department of Science and Technology, University of Sannio, via Francesco de Sanctis snc, Benevento 82100, Italy; ∥ Department of Chemistry, University of Rome Sapienza, P.le A.Moro 5, 00185 Rome, Italy

## Abstract

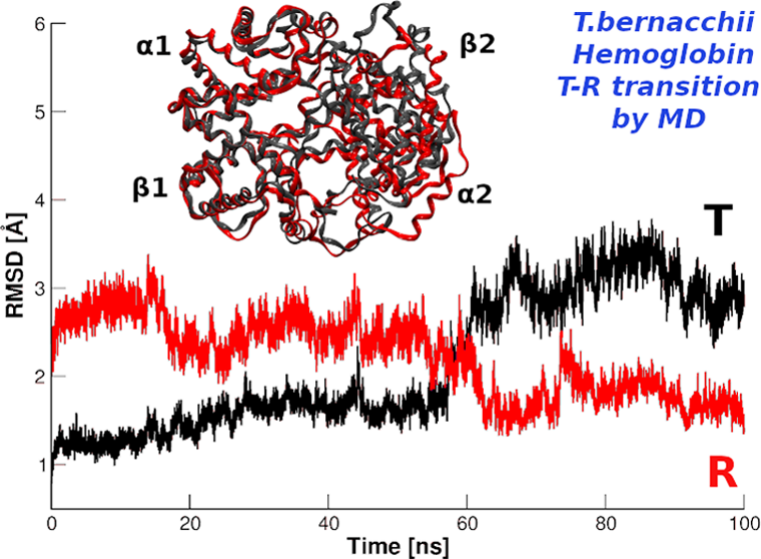

Tetrameric hemoglobins (Hbs) are prototypal systems for studies aimed at unveiling basic structure–function relationships as well as investigating the molecular/structural basis of adaptation of living organisms to extreme conditions. However, a chronological analysis of decade-long studies conducted on Hbs is illuminating on the difficulties associated with the attempts of gaining functional insights from static structures. Here, we applied molecular dynamics (MD) simulations to explore the functional transition from the T to the R state of the hemoglobin of the Antarctic fish *Trematomus bernacchii* (HbTb). Our study clearly demonstrates the ability of the MD technique to accurately describe the transition of HbTb from the T to R-like states, as shown by a number of global and local structural indicators. A comparative analysis of the structural states that HbTb assumes in the simulations with those detected in previous MD analyses conducted on HbA (human Hb) highlights interesting analogies (similarity of the transition pathway) and differences (distinct population of intermediate states). In particular, the ability of HbTb to significantly populate intermediate states along the functional pathway explains the observed propensity of this protein to assume these structures in the crystalline state. It also explains some functional data reported on the protein that indicate the occurrence of other functional states in addition to the canonical R and T ones. These findings are in line with the emerging idea that the classical two-state view underlying tetrameric Hb functionality is probably an oversimplification and that other structural states play important roles in these proteins. The ability of MD simulations to accurately describe the functional pathway in tetrameric Hbs suggests that this approach may be effectively applied to unravel the molecular and structural basis of Hbs exhibiting peculiar functional properties as a consequence of the environmental adaptation of the host organism.

## Introduction

In recent years, the field of protein structure prediction has been revolutionized by the development of machine-learning approaches.^1,2^ The application of these algorithms to the proteome of many species that are widely studied has largely expanded our knowledge of protein structures (see the EBI-AlphaFold Database at https://alphafold.ebi.ac.uk/). It is likely that in the near future, the amount of structural data will further increase with the application of these approaches to virtually all known protein sequences. Although the release of these structures will have a tremendous impact on our understanding of protein function, it is important to note that, in many cases, the definition of the precise functional implications of these static structural data will not be straightforward. In this context, a chronological analysis of studies conducted on vertebrate tetrameric hemoglobins (Hbs), which are prototype systems for elucidating structure–function relationships, is illuminating.^3−5^ Although the determination of the three-dimensional structure of these proteins, even in different binding states, dates back to the sixties,^6^ the elucidation of the structural mechanism underlying their functional transition is a long-standing issue that is still a topic of intense research activities. In this scenario, the discovery that all-atoms molecular dynamics (MD) simulations are able not only to emulate the transition from the T (tense) to the R (relaxed) crystallographic states but also to properly sample the intermediates that have been experimentally detected^7^ may represent a significant advance in the field.^8−13^

In addition to the classical questions related to the atomic-level forces that drive the transition from the oxygen low (T state) to the high (R state) affinity structures in response to physiological stimuli and to the origin of the Hb cooperativity, functional studies have highlighted a wide range of tetrameric Hb modifications as an effective tool for the adaptation needed to survive in specific environmental conditions. Among these, particularly intriguing are the studies devoted to unravel sequence/function and structure/function relationships in unusual environments such as birds flying at high altitudes^14,15^ or fishes living in extreme conditions as those adapted to survive in the Antarctic Ocean.^16,17^ Extensive functional studies on Antarctic fish Hbs (AntHbs) have highlighted a remarkable diversification in Hb functionality ranging from iceless fish, which lacks Hb,^18^ to fish containing multiple forms of this protein.^19−21^ AntHbs also display a variety of different properties as a consequence of external effectors. Although some AntHbs present pH-independent oxygen affinities, most of them are endowed with a strong Root effect.^22^ In vitro functional and structural investigations have unraveled some peculiar properties of these Hbs. In particular, AntHbs present a remarkable propensity to undergo oxidation with the ability of the iron to adopt a multitude of Fe(III) forms such as aquomet, hemichrome, and penta-coordinated states.^23^ Structural studies have shown that not only these states occur in the fully folded protein but they also frequently possess three-dimensional structures that perfectly fall in the T to R functional transition, thus providing a strong evidence of similarities between their unfolding and functional pathways.^7,24−29^ To provide structural explanations for the peculiar properties of these proteins and to check whether fully atomistic MD simulations can reproduce the functional transition in nonhuman tetrameric Hbs, we here report an extensive analysis of the Hb isolated from the Antarctic fish *Trematomus bernacchii* (HbTb). These MD simulations were validated by comparing the trajectory structures with crystallographic intermediate states identified for the closely related *Trematomus newnesi* hemoglobin (HbTn). Moreover, MD results were also compared to those recently obtained in similar MD studies conducted on human Hb (HbA).^12^ Our findings indicate that this approach can effectively describe the transition and also provide interesting analogies and differences between HbTb and human HbA.

## Results

### Overall Analysis of the T to R Transition of HbTb

**Figure 1:**
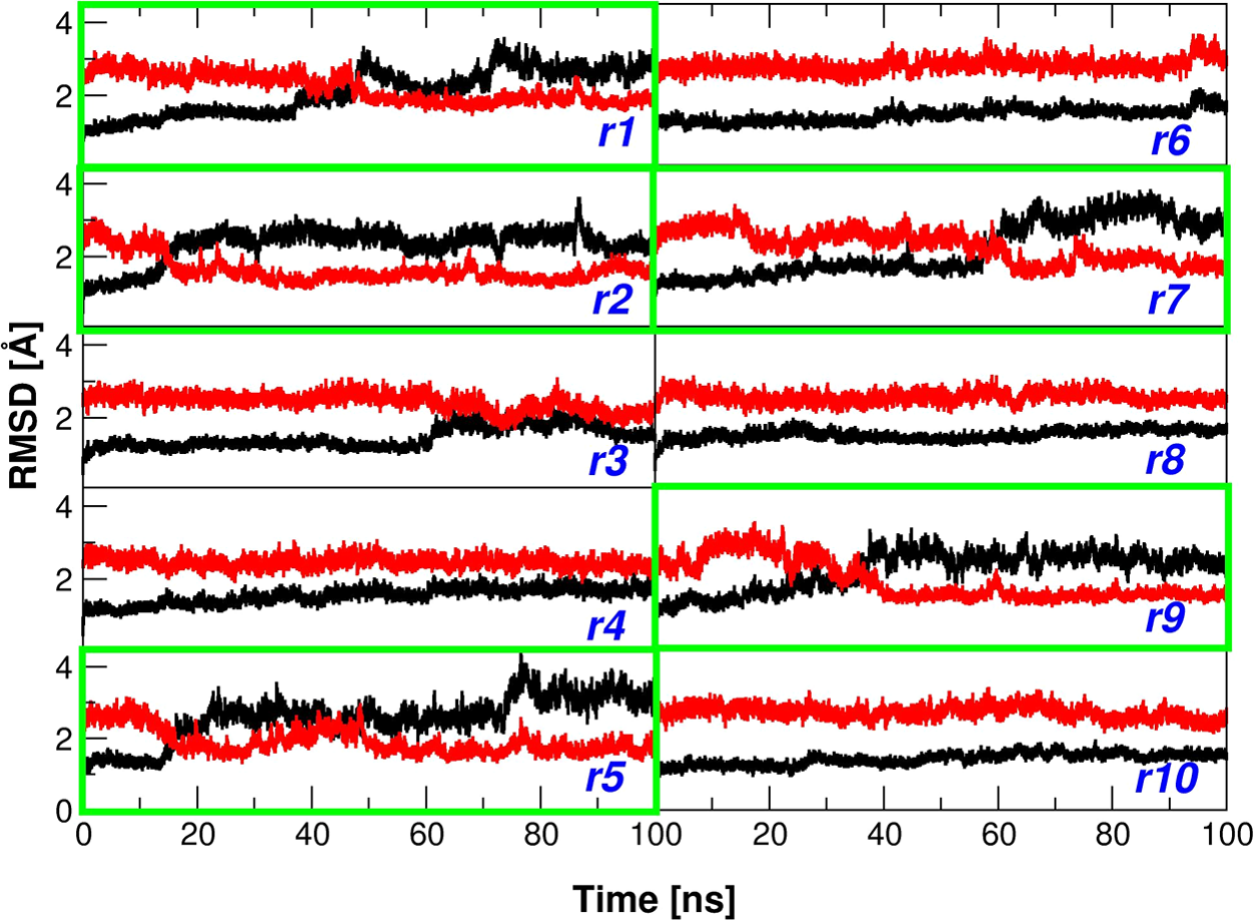
Root-mean-square deviation (RMSD) analysis. RMSD values (computed on C^α^ atoms) of the trajectory structures *versus* the starting T model (black, PDB ID: 2H8F) and the R state (red, PDB ID: 1PBX) in the HbTb simulations (runs r1 to r10). Green boxes indicate simulation runs with observed T → R transition.

Recent literature investigations have demonstrated that the functional transition of human Hb could be monitored by MD simulations starting from the tense T state. In these studies, the transition was facilitated by weakening a key interaction that stabilizes the tetramer in the T state, the electrostatic interaction between the side chains of Asp94β and the terminal His146β. By analogy, we here set up the simulations using as starting model the high-resolution structure of HbTb T state^26^ in which the carboxyl–carboxylate interaction (Asp95α1–Asp101β2) of the aspartic triad, which is believed to stabilize this state,^26,30^ was removed being Asp side chains deprotonated at physiological pH (see Methods for details). To perform an adequate sampling of the T state evolution, we performed 10 independent simulations (r1-r10). These were preliminarily analyzed by monitoring the root-mean-square deviation (RMSD) values of the trajectory structures *versus* the starting model (Figure 1). The inspection of the time evolution of these simulations indicates that in five of them (runs r1, r2, r5, r7, and r9), a transition was observed, and, importantly, the post-transition trajectory structures were much closer to the relaxed R state than to the starting T state. In other simulations (runs r4, r6, r8, and r10), no transition was observed. In the case of r3, an intermediate situation was detected, with structures of the second part of the trajectory displaying similar RMSD values when compared with either the R or the T state. To gain further insights into the time evolution of the HbTb T state, we compared the structures of the five trajectories that exhibit a clear transition with some crystallographic structures of vertebrate Hbs endowed with intermediate quaternary structures. In particular, we considered the well-characterized intermediate states (A, B,^7^ and H^28^) of the closely related HbTn, whose sequence presents only 13 amino acid mutations, out of 288 residues, compared to HbTb.^20^ The A and the B states, although structurally located in the T–R transition, present some similarities with the T state. On the other hand, the H state is a genuine intermediate state being clearly distinct from both the T and the R state. In addition to these states, we also considered the only intermediate state, although quite close to the R state, hitherto reported for HbA and denoted as HL-(C).^31^ As shown in Figure S1, trajectory structures when compared to the HL-(C) states essentially present RMSD values that are similar to those detected against the R state. Analogously, the RMSD values against the A and B states follow the trend observed for the T state. On the other hand, the RMSD trend against the H state presents an intermediate behavior between the R and the T state since it does not present major variations along the trajectories.

**Figure 2:**
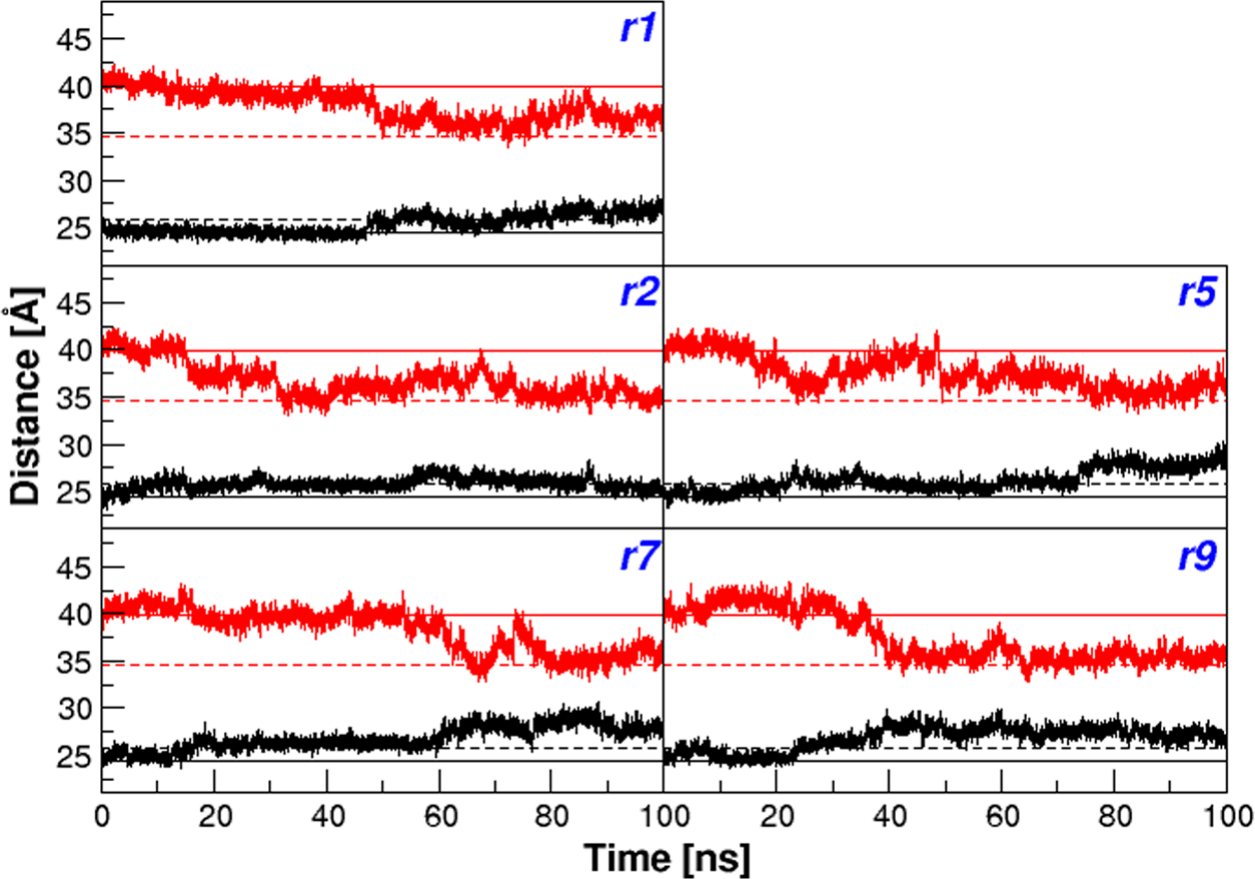
Iron–iron distances. Distances between the heme iron (Fe) atoms computed for the α1-β2 (black) and β1-β2 (red) dimers in the HbTb simulation runs with observed T→R transition. Correspondent Fe–Fe distance values detected in the crystallographic structures of the T and R state of HbTb are indicated by solid and dashed lines, respectively.

The observation reported in the previous paragraphs indicates that the structural transition observed in five distinct simulations may globally represent the functional transition associated with the oxygen binding of this protein. To substantiate this idea, we subsequently monitored the iron–iron distances in the trajectory structures as it is well established that in HbA, the T–R transition leads to an increase of the α1β2 and a concomitant decrease of the β1β2 iron–iron distance.^32−37^ Similar trends are detected in the crystal structures of HbTb.^21,26^ Indeed, comparing the T to the R state, a clear increase of the α1β2 (from 24.4 to 25.8 Å) and a decrease of the β1β2 (from 39.9 to 34.6 Å) distances are observed. As shown in Figure 2, the structures sampled in the final part of the simulations present larger iron–iron α1β2 and lower iron–iron β1β2 compared to those located at the beginning of the simulations. It is important to note that the largest variations of these distances (Figure 2) are concomitant with the structural transition detected in the RMSD diagrams (Figure 1). Collectively, these findings indicate that, at global level, the structural transition detected in five MD runs corresponds to the functional transition of these proteins.

### HbTb MD Simulation: Essential Dynamics Analysis

**Figure 3:**
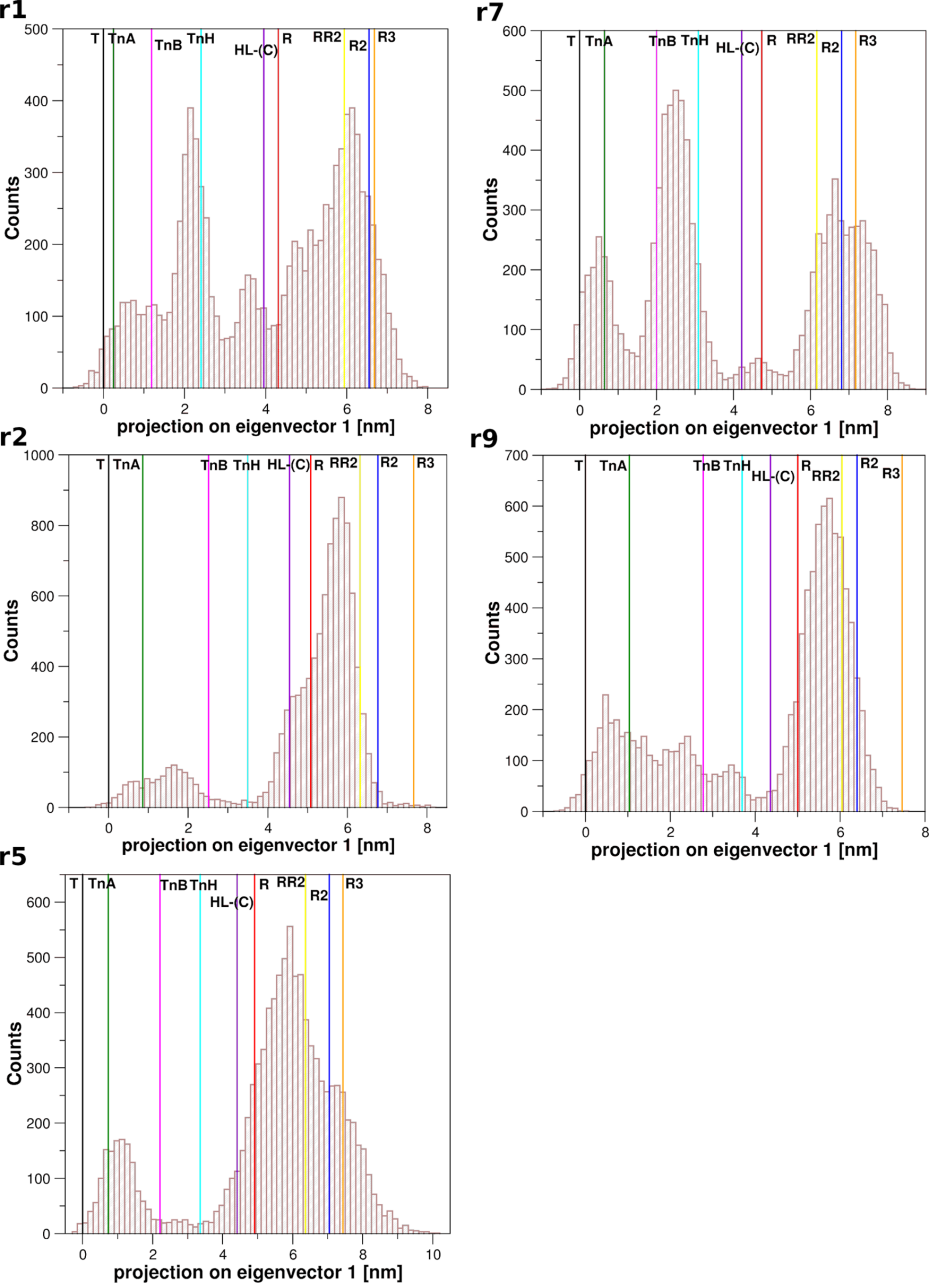
Essential dynamics analysis. Projection on the first eigenvector of the MD trajectories with observed T→R transition. The vertical solid lines correspond to the projections of the crystallographic structures of HbTb states: T (black, PDB ID: 2H8F) and R (red, PDB ID: 1PBX); HbTn intermediates: TnA (dark green, PDB ID: 5LFG), TnB (magenta, PDB ID: 5LFG), and TnH (cyan, PDB ID: 3D1K); HbA states: intermediate HL-(C) (violet, PDB ID: 4N7P), R2 (blue, PDB ID: 1BBB), RR2 (yellow, PDB ID: 1MKO), and R3 (orange, PDB ID: 4NI0).

To better characterize the T–R transition detected in the HbTb simulations described in the previous paragraphs, the trajectories r1, r2, r5, r7, and r9 were also analyzed by the essential dynamics (ED) method, where the principal motion directions of the molecular systems (i.e., the proteins) are represented by a set of eigenvectors (see Methods for further details). From the ensemble of the structures obtained from these trajectories, the eigenvectors were calculated and ranked according to their eigenvalues. Interestingly, for all of these simulations, the first principal component (eigenvector) accounts for most of the whole protein fluctuations (74–84%). On the basis of this finding, we projected the structures sampled by MD on the first eigenvector as well as the crystallographic structures of Antarctic fish Hbs corresponding to the end points of the transition (T and R states of HbTb) and to some intermediate states for both the individual runs (Figure 3) and for the concatenated trajectory in which the MD structures of the five simulations were collectively considered (Figure S2). In addition, we also projected some significant states of human HbA, including potential functional states (R2, RR2, and R3) that have been reported to fall beyond the classical T–R pathway.^5,33,35,36,38^

In line with the expectations, the crystallographic intermediate states fall within the pathway defined by the T and R states. In particular, the A state of HbTn and the HL-(C) state of HbA are quite close to the T and R states, respectively. On the other hand, the H state of HbTn (TnH) appears to be an intermediate state that is quite distinct from both the T and R structures. The position of the states R2, RR2, and R3 that fall beyond the transition further indicates the ability of this principal component to represent the functional motions of the protein.

In line with similar data collected for HbA,^12^ the projection of the trajectory structures of the HbTb simulations on this diagram highlights that they are frequently located beyond the R state and present similarities with the human R2, RR2, and R3 structures. This finding corroborates the hypotheses that these states play functional roles.^5,33,35,36,38^

A deep inspection of the distribution of the trajectory structures within the T and R states indicates that structural states presenting similarities with the H intermediate of HbTn are frequently populated (Figures 3 and S2). This is particularly evident in r1, r7, and r9 simulation runs (Figure 3).

This result is somehow surprising as MD simulations carried out on HbA using similar protocol and conditions resulted in sudden T to R transitions with a marginal population of the intermediate states.^12^

### HbTb MD Simulations: Monitoring of Specific Structural Probes of the Transition

Decade-long structural investigations carried out on vertebrate Hbs have highlighted a number of specific structural features that can be considered as fingerprints of either the R or the T state. With the aim of classifying the trajectory structures as a function of these specific structural parameters, we considered a number of indicators that were mutuated from studies conducted on HbA. In particular, we monitored the distance between the C^α^ atoms of the terminal His residues (His146) of the two β chains (C^α^ His146β1–C^α^ His146β2) that is very sensitive to the T to R transition and the key interactions that specifically stabilize the T state (i.e., Lys40α1 side chain–H146β2 COOH terminal group and Tyr42α1–Asp99β2 side chains).^12,32,39^ We also checked the distance between the residues located in the switch region α1CD-β2FG at the interface between the dimers α1β1 and α2β2 (C^α^ Ser44α1– C^α^ His97β2). In addition, we also considered some structural features that emerged from the analysis of crystallographic studies of AntHbs. In particular, the C^α^–C^α^ distance between proximal and distal His residues, which was found to significantly decrease in some intermediate states, and the distances among the side chains of the aspartic triad (Asp95α, Asp99β, and Asp101β).

**Figure 4:**
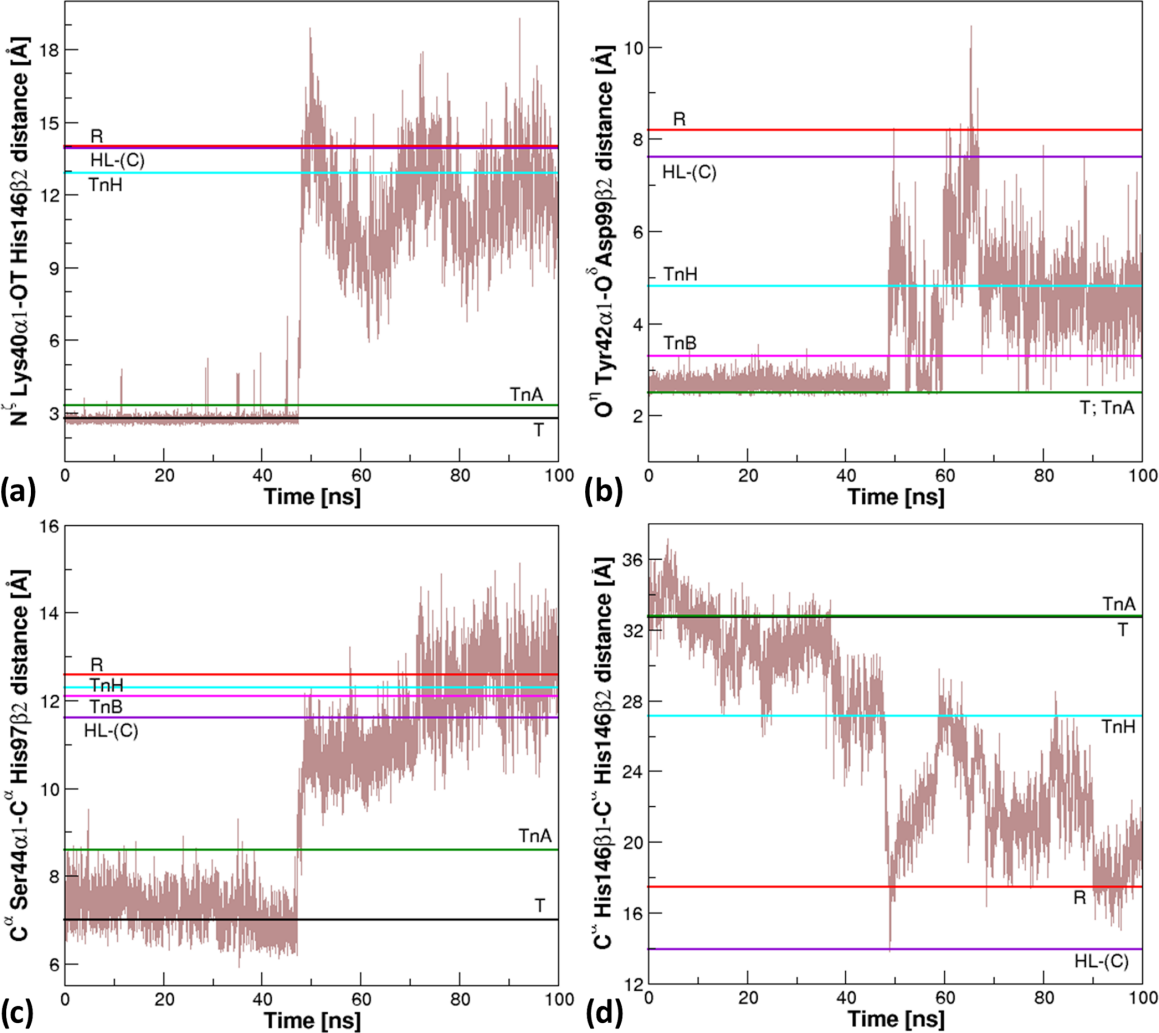
Time evolution of the structural probes that are characteristic of the different HbTb states in the r1 simulation run. Specifically, the distances (a) N^ζ^ Lys40α1–OT His146β2, (b) O^η^ Tyr42α1–O^δ^ Asp99β2, (c) C^α^ Ser44α1–C^α^ His97β2, and (d) C^α^ His146β1–C^α^ His146β2 are monitored.

As shown in Figures 4 and S3–S6 that report the evolution of the probes mutuated from HbA, the analysis of these specific parameters corroborates the indications arising from the global analysis. Indeed, the T–R transition is associated with the disappearance of the strong electrostatic interaction formed by the Lys40α1 side chain and the His146β2 COOH terminal group. Moreover, the analysis of the other probes clearly indicates that in a significant number of structures, these parameters assume the values observed in the HbTn states (see, for example, the distances C^α^ His146β1–C^α^ His146β2 and Tyr42α1–Asp99β2 side chains).

The C^α^–C^α^ distances between the distal and proximal His, whose shortening has been associated with intermediate states of the quaternary structure of AntHbs,^7,28^ do not highlight significant variations throughout the simulations (Figure S7). This implies that the transition does not require the major compression of the EF corner usually observed in crystallographic structures. Therefore, the closing of this region observed in these structures is due to the peculiar oxidation states of the iron (hemichrome, aquomet, oxidized penta-coordination).

**Figure 5:**
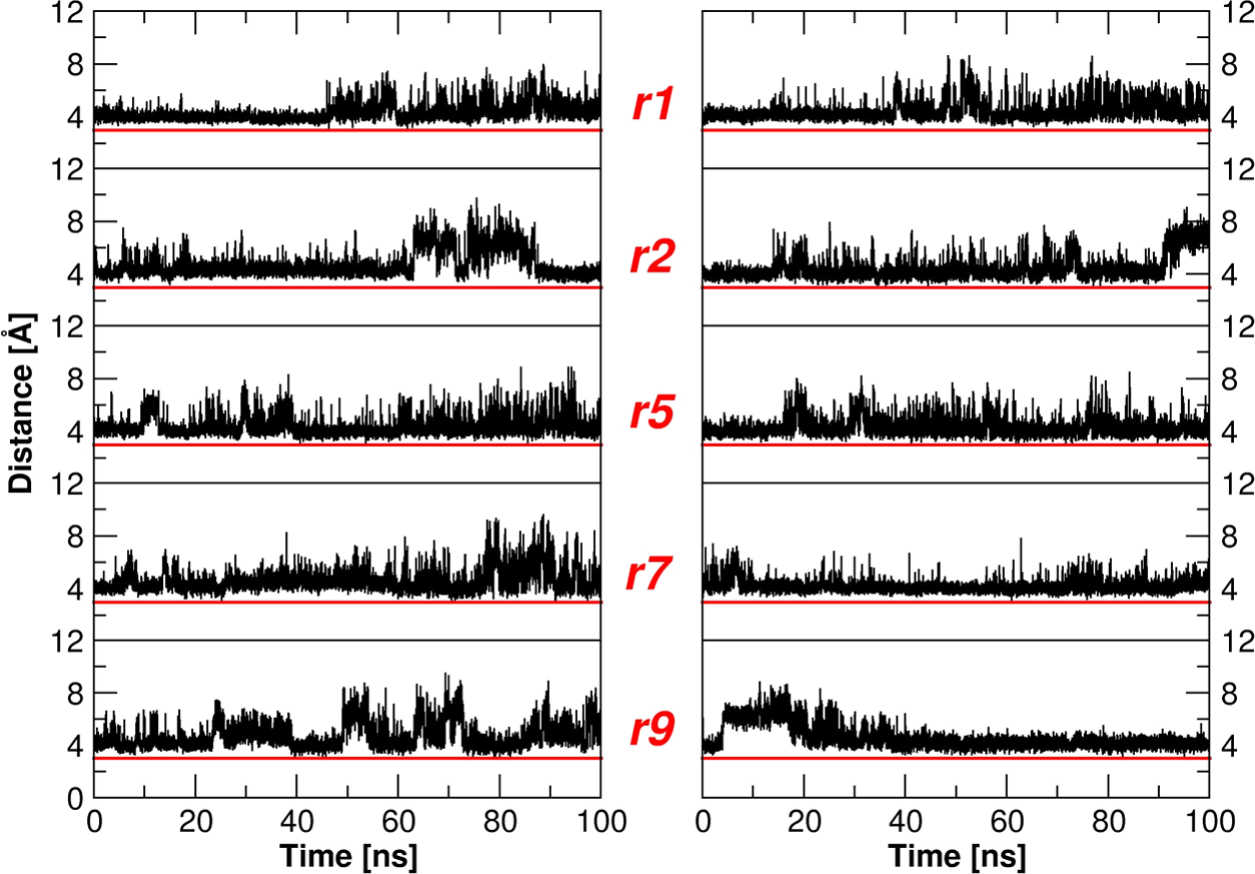
Inter-aspartic distances at α1β2 (left) and α2β1 (right) interfaces as observed along the MD trajctories. Minimum distances between Asp O^δ^ atoms of the pairs Asp95α1–Asp101β2 and Asp95α2–Asp101β1 are reported. Red line at 3.0 Å is given as an indicative H-bond length threshold.

**Figure 6:**
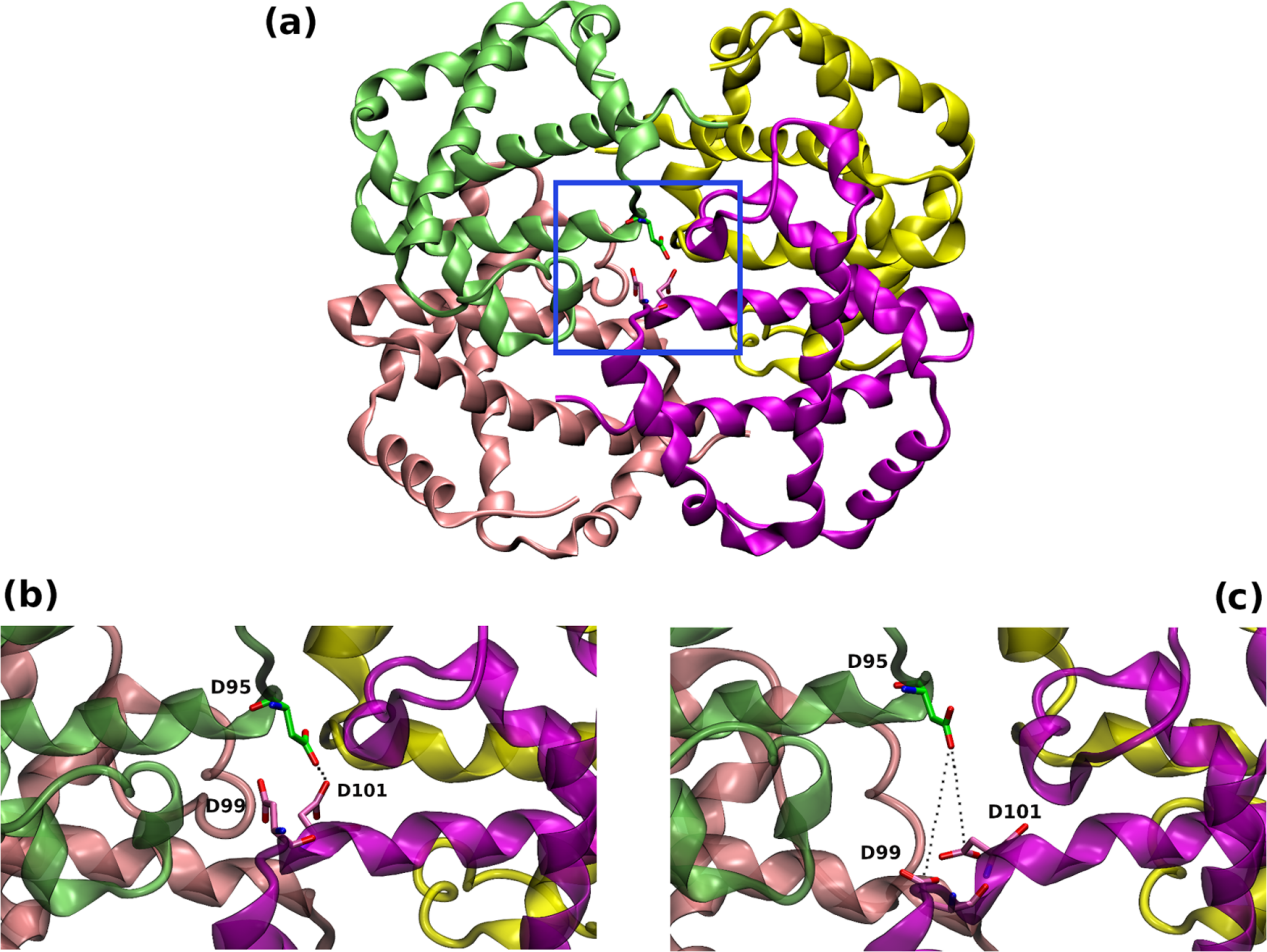
Cartoon representation of the three-dimensional structure of HbTb tetramer in the T state (PDB ID: 2H8F). (a) Asp95α1, Asp99β2, and Asp101β2 of the catalytic triad are shown as sticks. Close-up view of the α1β2 interfacing Asp residues (b) in the X-ray starting structure (PDB ID: 2H8F) and (c) in a representative trajectory frame (r2 simulation run, *t* = 75.5 ns) upon T → R transition. See also Figure 5.

As the carboxyl–carboxylate interaction formed by Asp95α1 and Asp101β2 (and Asp95α2-Asp101β1) of the catalytic triad is believed to be a key interaction that stabilizes the T state of HbTb at low pH values, thus producing the physiological strong dependence of the oxygen affinity of the protein as a function of the pH (Root effect^26,30^), we also monitored the evolution of these inter-aspartic distances along the MD trajectories (Figure 5). As shown in Figures 5 and 6, the distance between Asp95α1 and Asp101β2 (and Asp95α2 and Asp101β1) side chains is larger (with values spanning from ∼4 to ∼15 Å) than that found in the T state crystalline structure (2.5 Å) where the two residues form the carboxyl–carboxylate interaction. The distance between these residues often increases upon the T to R transition.

### Low-Temperature Hbtb MD Simulations

**Figure 7:**
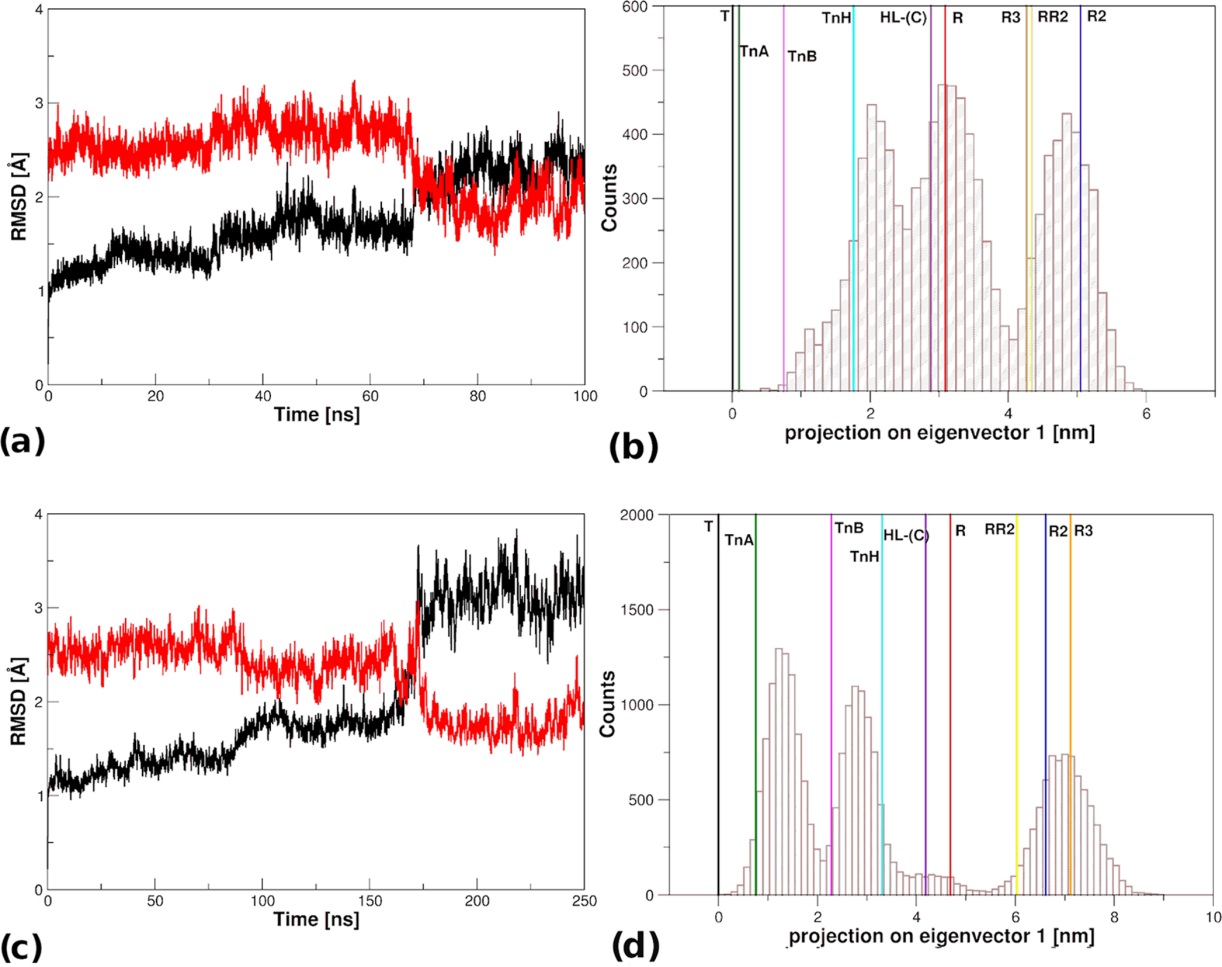
HbTb T–R transition at *T* = 273 K. RMSD values (computed on Cα atoms) of the trajectory structures versus the starting T model (black, PDB ID: 2H8F) and the R state (red, PDB ID: 1PBX) for (a) r1L and (b) r1L* simulations performed at 273 K. Projection of the MD trajectory on the first eigenvector for (c) r1L and (d) r1L*. The vertical solid lines correspond to the projections of the crystallographic structures of HbTb states: T (black, PDB ID: 2H8F) and R (red, PDB ID: 1PBX); HbTn intermediates: TnA (dark green, PDB ID: 5LFG), TnB (magenta, PDB ID: 5LFG), and TnH (cyan, PDB ID: 3D1K); HbA states: intermediate HL-(C) (violet, PDB ID: 4N7P), R2 (blue, PDB ID: 1BBB), RR2 (yellow, PDB ID: 1MKO), and R3 (orange, PDB ID: 4NI0).

The results illustrated in the previous paragraphs clearly indicate that HbTb undergoes a major structural transition during the simulations that recapitulates the variations associated with the conversion between its different functional states. One intriguing observation that emerged from these analyses is the significant population of intermediate states. It is important to note that the simulations as well as the crystallization experiments have been conducted at room temperature, which is quite different from the temperature of the Antarctic Ocean (∼ −1.8 °C) where this species lives.^40^ To verify whether the results obtained at room temperature were biased by the nonphysiological temperature (300 K) used in the simulations, these were repeated at 273 K (low-temperature simulations). In particular, ten 100 ns long independent runs were carried out. As shown in Figures 7a and S8, we observed the transition in a single run (r1_L_) only. Due to the low temperature used in this set of MD simulations, which might reflect a decrease in the transition rate, we also performed a longer (250 ns, r1_L_*) simulation, which indeed exhibited a clear T to R transition (Figure 7c).

The eigenvector analysis of the two low-temperature simulations in which the transition was observed clearly indicates that states resembling TnH were significantly populated (Figure 7b,d). This indication was also corroborated by the analysis of the specific probes (Figures S9–S10).

## Discussion

Tetrameric hemoglobins are prototypal systems for studies aimed at unveiling basic structure–function relationships.^6,41,42^ As these proteins are present in organisms living in extremely different environmental conditions, they are particularly suited also for studies focused on the identification of the basis of molecular adaptation. However, the rapid transition of these proteins from oxygen-bound to ligand-free states has hampered for decades a detailed structural characterization of their functional pathway, thus limiting the understanding of the underlying structure–function relationships. Indeed, the vast majority of crystallographic data available on tetrameric Hbs essentially represents variations of the R or the T state, with the valuable addition of the off-pathway structures R2, RR2, and R3.^5^ In this scenario, structural studies conducted on AntHbs constitute an intriguing exception as a significant portion of their crystallographic structures are structural intermediates of the canonical states. By exploiting the ability of all-atoms MD simulations to characterize at the atomic level the functional T–R transition of human HbA,^8,9,11,12^ we here applied this technique to the hemoglobin of the Antarctic fish *T. bernacchii* as MD simulations on AntHbs have been limited to the analysis of the behavior of isolated single chains.^43^ Our study, which, to the best of our knowledge, represents the first attempt to investigate the functional pathway of a nonhuman Hb by means of MD, clearly demonstrates the ability of this technique to accurately describe the transition of HbTb from T-like to R-like states, as demonstrated by a number of global and local structural indicators. The ED analysis also indicates that the starting T state not only does evolve toward R-like structures but also samples different conformational states, such as R2, RR2, and R3, which lie outside the T–R pathway.^5,33,35,36,38^ This finding suggests, in line with previous hypotheses, that the actual functional pathway of tetrameric Hbs also includes these states. It is worth underlining that a similar result has been obtained in MD simulations carried out on HbA.^12^ A comparative analysis of the structural states that HbTb sampled in the simulations with those detected in MD analyses conducted on HbA using a similar approach highlights interesting analogies and differences. In particular, the overall and the local structural features of the trajectory structures indicate that these proteins essentially follow a very similar pathway. In both cases, the ED analysis indicates that the first eigenvector is able to represent the vast majority of the protein motions. Moreover, the position of the crystallographic structures on the essential subspace provided by this eigenvector indicates that it essentially describes the motion direction underlying the T–R transition. These similarities clearly indicate that atomic-level features of the functional switch of these Hbs are well conserved, despite the hundredths of millions of years that evolutionarily separate these proteins.

However, although the two proteins follow the same structural pathway in the simulations, a comparative analysis indicates that some intermediate states detected in the crystallographic studies on tetrameric Hbs, notably tetramer H of HbTn, that are barely detected in the HbA MD simulations^12^ are frequently well populated in HbTb simulations (Figure 3). Our data suggest that the interconversion between the T and the R state, which is very rapid in HbA, is somehow smoothed in HbTb. This finding provides a clear explanation to the puzzling observation that intermediate states are frequently detected for AntHb, while they are extremely elusive for HbA, despite the several hundredths of independent structures reported for this protein in the PDB.^39^ In this scenario, the larger population of these states in AntHb compared to HbA has allowed their trapping in the crystalline state and the visualization of a variety of peculiar and partially oxidized states that are not compatible with the canonical R and T states. Moreover, the present results perfectly fit with the observation that in HbTb, a significant degree of oxygen-binding cooperativity can be ascribed to tertiary conformational changes toward R-like positions that anticipate the real T–R quaternary transition.^44^ In general terms, these findings are in line with the emerging idea that the classical two-state view underlying tetrameric Hb functionality is probably an oversimplification and that other structural states play important roles in these proteins.^45^

The ability of MD simulations to accurately describe the functional pathway in both HbA and Hb isolated from an organism living in extreme conditions strongly suggests that this approach may be effectively applied to unravel the molecular and structural basis of Hbs exhibiting peculiar functional properties, as a consequence of the environmental adaptation of the host organism.

In conclusion, the historical analysis of the controversial and several decade-long investigations aimed at describing Hb functionality at atomic level perfectly illustrates the difficulties associated with gaining functional insights from static structures. The present study demonstrates that the application of current standard protocols may provide important information in this field. This finding is particularly important in the AlphaFold era, in which a huge amount of structural data should be appropriately exploited to gain biologically relevant insights.

## Methods

### Structural Models

Fully atomistic MD simulations were performed on the T state of HbTb. In detail, the transition from this form to the R state was studied at neutral pH using the high-resolution crystallographic structure of the tetrameric TbHb in the deoxygenated form determined at pH 6.2 (PDB ID: 2H8F)^26^ as starting model. The protonation states of the histidine residues were chosen following the protocol adopted for HbA in Balasco et al.^12^ In detail, for conserved His between HbA and HbTb, the same protonation state of HbA was adopted also for HbTb, whereas the state of the additional His residues of HbTb was assigned by GROMACS software. As done for HbA,^8−11^ to favor the T–R transition, the terminal His of the β chains were kept in the deprotonated state. Moreover, to emulate pH conditions close to neutrality, the three Asp residues (Asp95α, Asp99β, and Asp101β) of the aspartic triad were kept deprotonated (Asp side-chain p*K*_a_ ∼ 3.9).

To analyze the overall and local structural features of the trajectory models, we considered a number of both canonical and putative states identified for HbA,^31^ for HbTb, and for the hemoglobins from the Antarctic *T. newnesi* (HbTn) fish (96% sequence identity) whose intermediate states along the functional transition have been well-characterized.^7,28^ Coordinates of these structural models were retrieved from the Protein Data Bank (https://www.rcsb.org/). In detail, for HbTb, the canonical T (PDB ID: 2H8F)^26^ and R (PDB ID: 1PBX)^21^ states were considered. For HbA the following PDB structures were used: 1BBB (R2 state),^33^1MKO (RR2 state),^35^4NI0 (R3 state),^46^ and 4N7P(31) (intermediate half-liganded HL-(C) state). The structures of HbTn tetramers A, B^7^ (TnA and TnB, PDB ID: 5LFG), and H^28^ (TnH, PDB ID: 3D1K) were also considered. Structural details of these crystallographic models are reported in Table S1.

### Protocol

The GROMACS^47^ software (version 2020.3) has been used to carry out fully atomistic MD simulations of the HbTb T state using the CHARMM-36^48^ all-atom force field. The protein model was solvated with water molecules of the TIP3P model^49^ in a cubic box of 150 Å edge size. The system was neutralized with sodium and chloride counterions to achieve a salt concentration of 0.15 mol/L. Electrostatic interactions were treated with the particle-mesh Ewald (PME) method^50^ (1 Å grid spacing and 10^–6^ relative tolerance), whereas a 10 Å switching was considered for the Lennard–Jones (LJ) interactions. The LINCS algorithm^51^ was used to constrain bond lengths. The system was energy minimized in 50000 steps using the steepest descent algorithm. Then, it was equilibrated in two phases. First, the system temperature was raised to 300 K in 300 ps using increments of 10 K (NVT). The system pressure was then equilibrated at 1 atm in 500 ps (NpT). The Velocity Rescaling and Parrinello–Rahman algorithms^52,53^ were applied for temperature and pressure control, respectively.

MD production runs were performed at the constant pressure of 1 atm and at two different temperatures with a time step of 2 fs. In detail, 10 independent replicas of 100 ns were performed at 300 K (r1–r10) and 273 K (r1_L_–r10_L_) with random initial velocities (gen_seed option in mdp input file). In addition, we run a longer simulation (250 ns) to improve the conformational sampling at T = 273 K (r1_L_*).

We apply the essential dynamics^54^ technique to carry out the principal component analysis of the MD runs. In detail, we built the covariance matrix of the protein C^α^ atomic positions. The diagonalization of this matrix provides a set of eigenvectors with their associated eigenvalues that represent the principal protein motions. Using this approach, it is possible to represent the protein overall dynamics in a reduced essential subspace described by the first eigenvectors, which are therefore defined as principal components. The gmx covar and gmx anaeig tools of GROMACS software were used to build the covariance matrix and to calculate the projection with respect to the first eigenvector.

### Data and Software Availability

Coordinates of the three-dimensional structures used in this project were retrieved from the Protein Data Bank (https://www.rcsb.org/). MD simulations were performed using GROMACS^47^ software (version 2020.3). GROMACS tools and the Visual Molecular Dynamics (VMD) program^55^ were used to conduct the structural analyses of trajectory models. Figures were generated using the VMD and Xmgrace software version 50125 (https://plasma-gate.weizmann.ac.il/Grace/). Authors will release MD trajectories upon article publication using Zenodo repository (https://zenodo.org/).

## Supporting Information Available

Root-mean-square deviation (RMSD) analysis of the HbTb simulation runs with observed T → R transition (Figure S1); essential dynamics analysis on the concatenated MD trajectories with observed T → R transition (Figure S2); time evolution of the structural probes that are characteristic of the different HbTb states in simulation runs with observed T → R transition (Figures S3–S6); distributions of the C^α^–C^α^ distance between the distal and proximal His residues in α1, α2 (His58-His87) and β1, β2 (His63-His92) chains in MD runs with observed T → R transition (Figure S7); root-mean-square deviation (RMSD) analysis of the HbTb simulations carried at *T* = 273 K (Figure S8); time evolution of the structural probes that are characteristic of the different HbTb states in r1_L_ and r1_L_* simulation runs at *T* = 273 K (Figures S9–S10); and details of representative crystallographic structures of HbTb (T and R), HbA (R2, RR2, R3, and HL-(C)), and HbTn (TnA, TnB, and TnH) (Table S1) (PDF)

## Abbreviations

Hb: hemoglobin
HbA: human hemoglobin
AntHbs: Hbs isolated from Antarctic fish
HbTb: Hb from *T. bernacchii*
HbTn: Hb from *T. newnesi*
TnA: Hb tetramer A from *T. newnesi*
TnB: Hb tetramer B from *T. newnesi*
TnH: Hb tetramer H from *T. newnesi*
PDB: protein data bank
MD: molecular dynamics
RMSD: root-mean-square deviation
ED: essential dynamics

## References

[ref1] Senior A. W.; Evans R.; Jumper J.; Kirkpatrick J.; Sifre L.; Green T.; Qin C.; Žídek A.; Nelson A. W. R.; Bridgland A.; Penedones H.; Petersen S.; Simonyan K.; Crossan S.; Kohli P.; Jones D. T.; Silver D.; Kavukcuoglu K.; Hassabis D. Improved protein structure prediction using potentials from deep learning. Nature 2020, 577, 706–710. 10.1038/s41586-019-1923-7. 31942072

[ref2] Tunyasuvunakool K.; Adler J.; Wu Z.; Green T.; Zielinski M.; Žídek A.; Bridgland A.; Cowie A.; Meyer C.; Laydon A.; Velankar S.; Kleywegt G. J.; Bateman A.; Evans R.; Pritzel A.; Figurnov M.; Ronneberger O.; Bates R.; Kohl S. A. A.; Potapenko A.; Ballard A. J.; Romera-Paredes B.; Nikolov S.; Jain R.; Clancy E.; Reiman D.; Petersen; Senior A. W.; Kavukcuoglu K.; Birney E.; Kohli P.; Jumper J.; Hassabis D. Highly accurate protein structure prediction for the human proteome. Nature 2021, 596, 590–596. 10.1038/s41586-021-03828-1. 34293799 PMC8387240

[ref3] Olson J. S. Lessons Learned from 50 Years of Hemoglobin Research: Unstirred and Cell-Free Layers, Electrostatics, Baseball Gloves, and Molten Globules. Antioxid. Redox Signaling 2020, 32, 228–246. 10.1089/ars.2019.7876. PMC6948003 31530172

[ref4] Brunori M. Hemoglobin is an honorary enzyme. Trends Biochem. Sci. 1999, 24, 158–161. 10.1016/S0968-0004(99)01380-8. 10322423

[ref5] Ahmed M. H.; Ghatge M. S.; Safo M. K. Hemoglobin: Structure, Function and Allostery. In Vertebrate and Invertebrate Respiratory Proteins, Lipoproteins and other Body Fluid Proteins; Hoeger U.; Harris J. R., Eds.; Springer International Publishing, 2020; Vol. 94, pp 345–382. 10.1007/978-3-030-41769-7_14 PMC7370311 32189307

[ref6] Perutz M. F. Stereochemistry of Cooperative Effects in Haemoglobin: Haem–Haem Interaction and the Problem of Allostery. Nature 1970, 228, 726–734. 10.1038/228726a0. 5528785

[ref7] Vitagliano L.; Mazzarella L.; Merlino A.; Vergara A. Fine Sampling of the R→T Quaternary-Structure Transition of a Tetrameric Hemoglobin. Chem. - Eur. J. 2017, 23, 605–613. 10.1002/chem.201603421. 27808442

[ref8] Hub J. S.; Kubitzki M. B.; de Groot B. L. Spontaneous Quaternary and Tertiary T-R Transitions of Human Hemoglobin in Molecular Dynamics Simulation. PLoS Comput. Biol. 2010, 6, e1000774 10.1371/journal.pcbi.1000774. 20463873 PMC2865513

[ref9] Vesper M. D.; de Groot B. L. Collective Dynamics Underlying Allosteric Transitions in Hemoglobin. PLoS Comput. Biol. 2013, 9, e1003232 10.1371/journal.pcbi.1003232. 24068910 PMC3777908

[ref10] El Hage K.; Hédin F.; Gupta P. K.; Meuwly M.; Karplus M. Valid molecular dynamics simulations of human hemoglobin require a surprisingly large box size. eLife 2018, 7, e35560 10.7554/eLife.35560. 29998846 PMC6042964

[ref11] Gapsys V.; de Groot B. L. On the importance of statistics in molecular simulations for thermodynamics, kinetics and simulation box size. eLife 2020, 9, e57589 10.7554/eLife.57589. 32812868 PMC7481008

[ref12] Balasco N.; Alba J.; D’Abramo M.; Vitagliano L. Quaternary Structure Transitions of Human Hemoglobin: An Atomic-Level View of the Functional Intermediate States. J. Chem. Inf. Model. 2021, 61, 3988–3999. 10.1021/acs.jcim.1c00315. 34375114 PMC9473481

[ref13] Wodak S. J.; Paci E.; Dokholyan N. V.; Berezovsky I. N.; Horovitz A.; Li J.; Hilser V.; Bahar I.; Karanicolas J.; Stock G.; Hamm P.; Stote R. H.; Eberhardt J.; Chebaro Y.; Dejaegere A.; Cecchini M.; Changeux J. P.; Bolhuis P. G.; Vreede J.; Faccioli P.; Orioli S.; Ravasio R.; Yan L.; Brito C.; Wyart M.; Gkeka P.; Rivalta I.; Palermo G.; McCammon J. A.; Panecka-Hofman J.; Wade R. C.; Di Pizio A.; Niv M. Y.; Nussinov R.; Tsai C. J.; Jang H.; Padhorny D.; Kozakov D.; McLeish T. Allostery in Its Many Disguises: From Theory to Applications. Structure 2019, 27, 566–578. 10.1016/j.str.2019.01.003. 30744993 PMC6688844

[ref14] Storz J. F.; Moriyama H. Mechanisms of Hemoglobin Adaptation to High Altitude Hypoxia. High Alt. Med. Biol. 2008, 9, 148–157. 10.1089/ham.2007.1079. 18578646 PMC3140315

[ref15] Natarajan C.; Jendroszek A.; Kumar A.; Weber R. E.; Tame J. R. H.; Fago A.; Storz J. F. Molecular basis of hemoglobin adaptation in the high-flying bar-headed goose. PLoS Genet. 2018, 14, e1007331 10.1371/journal.pgen.1007331. 29608560 PMC5903655

[ref16] Bargelloni L.; Marcato S.; Patarnello T. Antarctic fish hemoglobins: Evidence for adaptive evolution at subzero temperature. Proc. Natl. Acad. Sci. U.S.A. 1998, 95, 8670–8675. 10.1073/pnas.95.15.8670. 9671736 PMC21134

[ref17] Giordano D.; Pesce A.; Boechi L.; Bustamante J. P.; Caldelli E.; Howes B. D.; Riccio A.; di Prisco G.; Nardini M.; Estrin D.; Smulevich G.; Bolognesi M.; Verde C. Structural flexibility of the heme cavity in the cold-adapted truncated hemoglobin from the Antarctic marine bacterium *Pseudoalteromonas haloplanktis* TAC125. FEBS J. 2015, 282, 2948–2965. 10.1111/febs.13335. 26040838

[ref18] Feller G.; Gerday C. Adaptations of the hemoglobinless Antarctic icefish (Channichthyidae) to hypoxia tolerance. Comp. Biochem. Physiol., Part A: Physiol. 1997, 118, 981–987. 10.1016/S0300-9629(97)86786-2.

[ref19] di Prisco G.; Carratore V.; Cocca E.; Riccio A.; Tamburrini M. Molecular structure and functional adaptations of hemoglobins from Antarctic marine organisms. Ital. J. Zool. 2000, 67, 37–46. 10.1080/11250000009356354.

[ref20] D’Avino R.; Caruso C.; Tamburrini M.; Romano M.; Rutigliano B.; Polverino de Laureto P.; Camardella L.; Carratore V.; di Prisco G. Molecular characterization of the functionally distinct hemoglobins of the Antarctic fish *Trematomus newnesi*. J. Biol. Chem. 1994, 269, 9675–9681. 10.1016/S0021-9258(17)36935-1. 8144556

[ref21] Camardella L.; Caruso C.; D’Avino R.; di Prisco G.; Rutigliano B.; Tamburrini M.; Fermi G.; Perutz M. F. Haemoglobin of the Antarctic fish Pagothenia bernacchii. J. Mol. Biol. 1992, 224, 449–460. 10.1016/0022-2836(92)91007-C. 1560461

[ref22] Verde C.; Vergara A.; Giordano D.; Mazzarella L.; Di Prisco G. The Root effect – a structural and evolutionary perspective. Antarct. Sci. 2007, 19, 271–278. 10.1017/S095410200700034X.

[ref23] Vitagliano L.; Bonomi G.; Riccio A.; di Prisco G.; Smulevich G.; Mazzarella L. The oxidation process of Antarctic fish hemoglobins. Eur. J. Biochem. 2004, 271, 1651–1659. 10.1111/j.1432-1033.2004.04054.x. 15096204

[ref24] Riccio A.; Vitagliano L.; di Prisco G.; Zagari A.; Mazzarella L. The crystal structure of a tetrameric hemoglobin in a partial hemichrome state. Proc. Natl. Acad. Sci. U.S.A. 2002, 99, 9801–9806. 10.1073/pnas.132182099. 12093902 PMC125021

[ref25] Mazzarella L.; D’Avino R.; di Prisco G.; Savino C.; Vitagliano L.; Moody P. C. E.; Zagari A. Crystal structure of *Trematomus newnesi* haemoglobin re-opens the root effect question. J. Mol. Biol. 1999, 287, 897–906. 10.1006/jmbi.1999.2632. 10222199

[ref26] Mazzarella L.; Vergara A.; Vitagliano L.; Merlino A.; Bonomi G.; Scala S.; Verde C.; di Prisco G. High resolution crystal structure of deoxy hemoglobin from *Trematomus bernacchii* at different pH values: The role of histidine residues in modulating the strength of the root effect. Proteins 2006, 65, 490–498. 10.1002/prot.21114. 16909420

[ref27] Merlino A.; Vitagliano L.; Howes B. D.; Verde C.; di Prisco G.; Smulevich G.; Sica F.; Vergara A. Combined crystallographic and spectroscopic analysis of *Trematomus bernacchii* hemoglobin highlights analogies and differences in the peculiar oxidation pathway of Antarctic fish hemoglobins. Biopolymers 2009, 91, 1117–1125. 10.1002/bip.21206. 19373928

[ref28] Vitagliano L.; Vergara A.; Bonomi G.; Merlino A.; Verde C.; di Prisco G.; Howes B. D.; Smulevich G.; Mazzarella L. Spectroscopic and Crystallographic Characterization of a Tetrameric Hemoglobin Oxidation Reveals Structural Features of the Functional Intermediate Relaxed/Tense State. J. Am. Chem. Soc. 2008, 130, 10527–10535. 10.1021/ja803363p. 18642904

[ref29] Vergara A.; Franzese M.; Merlino A.; Vitagliano L.; Verde C.; di Prisco G.; Lee H. C.; Peisach J.; Mazzarella L. Structural Characterization of Ferric Hemoglobins from Three Antarctic Fish Species of the Suborder Notothenioidei. Biophys. J. 2007, 93, 2822–2829. 10.1529/biophysj.107.105700. 17545238 PMC1989692

[ref30] Ito N.; Komiyama N. H.; Fermi G. Structure of Deoxyhaemoglobin of the Antarctic Fish Pagothenia bernacchii with an Analysis of the Structural Basis of the Root Effect by Comparison of the Liganded and Unliganded Haemoglobin Structures. J. Mol. Biol. 1995, 250, 648–658. 10.1006/jmbi.1995.0405. 7623382

[ref31] Shibayama N.; Sugiyama K.; Tame J. R. H.; Park S. Y. Capturing the Hemoglobin Allosteric Transition in a Single Crystal Form. J. Am. Chem. Soc. 2014, 136, 5097–5105. 10.1021/ja500380e. 24635037

[ref32] Baldwin J.; Chothia C. Haemoglobin: The structural changes related to ligand binding and its allosteric mechanism. J. Mol. Biol. 1979, 129, 175–220. 10.1016/0022-2836(79)90277-8. 39173

[ref33] Silva M. M.; Rogers P. H.; Arnone A. A third quaternary structure of human hemoglobin A at 1.7-A resolution. J. Biol. Chem. 1992, 267, 17248–17256. 10.1016/S0021-9258(18)41919-9. 1512262

[ref34] Lukin J. A.; Kontaxis G.; Simplaceanu V.; Yuan Y.; Bax A.; Ho C. Quaternary structure of hemoglobin in solution. Proc. Natl. Acad. Sci. U.S.A. 2003, 100, 517–520. 10.1073/pnas.232715799. 12525687 PMC141027

[ref35] Safo M. K.; Abraham D. J. The Enigma of the Liganded Hemoglobin End State: A Novel Quaternary Structure of Human Carbonmonoxy Hemoglobin. Biochemistry 2005, 44, 8347–8359. 10.1021/bi050412q. 15938624

[ref36] Jenkins J. D.; Musayev F. N.; Danso-Danquah R.; Abraham D. J.; Safo M. K. Structure of relaxed-state human hemoglobin: insight into ligand uptake, transport and release. Acta Crystallogr., Sect. D: Biol. Crystallogr. 2009, 65, 41–48. 10.1107/S0907444908037256. 19153465

[ref37] Safo M. K.; Ahmed M. H.; Ghatge M. S.; Boyiri T. Hemoglobin–ligand binding: Understanding Hb function and allostery on atomic level. Biochim. Biophys. Acta, Proteins Proteomics 2011, 1814, 797–809. 10.1016/j.bbapap.2011.02.013. 21396487

[ref38] Tame J. R. H. What is the true structure of liganded haemoglobin?. Trends Biochem. Sci. 1999, 24, 372–377. 10.1016/S0968-0004(99)01444-9. 10500299

[ref39] Balasco N.; Vitagliano L.; Merlino A.; Verde C.; Mazzarella L.; Vergara A. The unique structural features of carbonmonoxy hemoglobin from the sub-Antarctic fish Eleginops maclovinus. Sci. Rep. 2019, 9, 18987 10.1038/s41598-019-55331-3. 31831781 PMC6908587

[ref40] Kock K.-H.; Everson I. Age, Growth and Maximum Size of Antarctic Notothenioid Fish — Revisited. In Fishes of Antarctica; Springer: Milan, 1998; pp 29–40.

[ref41] Miele A. E.; Bellelli A.; Brunori M. Hemoglobin Allostery: New Views on Old Players. J. Mol. Biol. 2013, 425, 1515–1526. 10.1016/j.jmb.2012.12.018. 23274140

[ref42] Pillai A. S.; Chandler S. A.; Liu Y.; Signore A. V.; Cortez-Romero C. R.; Benesch J. L. P.; Laganowsky A.; Storz J. F.; Hochberg G. K. A.; Thornton J. W. Origin of complexity in haemoglobin evolution. Nature 2020, 581, 480–485. 10.1038/s41586-020-2292-y. 32461643 PMC8259614

[ref43] Merlino A.; Vergara A.; Sica F.; Aschi M.; Amadei A.; Di Nola A.; Mazzarella L. Free-Energy Profile for CO Binding to Separated Chains of Human and *Trematomus newnesi* Hemoglobin: Insights from Molecular Dynamics Simulations and Perturbed Matrix Method. J. Phys. Chem. B 2010, 114, 7002–7008. 10.1021/jp908525s. 20433181

[ref44] Ronda L.; Merlino A.; Bettati S.; Verde C.; Balsamo A.; Mazzarella L.; Mozzarelli A.; Vergara A. Role of tertiary structures on the Root effect in fish hemoglobins. Biochim. Biophys. Acta 2013, 1834, 1885–1893. 10.1016/j.bbapap.2013.01.031. 23376186

[ref45] Shibayama N. Allosteric transitions in hemoglobin revisited. Biochim. Biophys. Acta 2020, 1864, 129335 10.1016/j.bbagen.2019.03.021. 30951803

[ref46] Nakagawa A.; Lui F. E.; Wassaf D.; Yefidoff-Freedman R.; Casalena D.; Palmer M. A.; Meadows J.; Mozzarelli A.; Ronda L.; Abdulmalik O.; Bloch K. D.; Safo M. K.; Zapol W. M. Identification of a small molecule that increases hemoglobin oxygen affinity and reduces SS erythrocyte sickling. ACS Chem. Biol. 2014, 9, 2318–2325. 10.1021/cb500230b. 25061917 PMC4205001

[ref47] Van Der Spoel D.; Lindahl E.; Hess B.; Groenhof G.; Mark A. E.; Berendsen H. J. C. GROMACS: Fast, flexible, and free. J. Comput. Chem. 2005, 26, 1701–1718. 10.1002/jcc.20291. 16211538

[ref48] Lee J.; Cheng X.; Swails J. M.; Yeom M. S.; Eastman P. K.; Lemkul J. A.; Wei S.; Buckner J.; Jeong Y. C.; Qi Y.; Jo S.; Pande V. S.; Case D. A.; Brooks C. L.; MacKerell A. D.; Klauda J. B.; Im W. CHARMM-GUI Input Generator for NAMD, GROMACS, AMBER, OpenMM, and CHARMM/OpenMM Simulations Using the CHARMM36 Additive Force Field. J. Chem. Theory Comput. 2016, 12, 405–413. 10.1021/acs.jctc.5b00935. 26631602 PMC4712441

[ref49] Jorgensen W. L.; Chandrasekhar J.; Madura J. D.; Impey R. W.; Klein M. L. Comparison of simple potential functions for simulating liquid water. J. Chem. Phys. 1983, 79, 926–935. 10.1063/1.445869.

[ref50] Darden T.; York D.; Pedersen L. Particle mesh Ewald: An *N* ·log(*N*) method for Ewald sums in large systems. J. Chem. Phys. 1993, 98, 10089–10092. 10.1063/1.464397.

[ref51] Hess B.; Bekker H.; Berendsen H. J. C.; Fraaije J. G. E. M. LINCS: A linear constraint solver for molecular simulations. J. Comput. Chem. 1997, 18, 1463–1472. 10.1002/(SICI)1096-987X(199709)18:12<1463::AID-JCC4>3.0.CO;2-H.

[ref52] Bussi G.; Donadio D.; Parrinello M. Canonical sampling through velocity rescaling. J. Chem. Phys. 2007, 126, 014101 10.1063/1.2408420. 17212484

[ref53] Parrinello M.; Rahman A. Polymorphic transitions in single crystals: A new molecular dynamics method. J. Appl. Phys. 1981, 52, 7182–7190. 10.1063/1.328693.

[ref54] Amadei A.; Linssen A. B. M.; Berendsen H. J. C. Essential dynamics of proteins. Proteins 1993, 17, 412–425. 10.1002/prot.340170408. 8108382

[ref55] Humphrey W.; Dalke A.; Schulten K. VMD: visual molecular dynamics. J. Mol. Graphics 1996, 14, 33–38. 10.1016/0263-7855(96)00018-5. 8744570

