# Supplementary material for: Atomic-Level View of the Functional Transition in Vertebrate Hemoglobins: The Case of Antarctic Fish Hbs
Source: J Chem Inf Model. 2022 Aug 5;62(16):3874–84. doi: 10.1021/acs.jcim.2c00727 (PMC9400108; doi:10.1021/acs.jcim.2c00727)
Supplement: Supplementary file 1 — ci2c00727_si_001.pdf [file ci2c00727_si_001.pdf]

Supporting Information

Atomic-Level View of the Functional Transition in Vertebrate Hemoglobins: The Case of Antarctic Fish Hbs

Nicole Balasco[†], Antonella Paladino[*‡], Giuseppe Graziano[§], Marco D'Abramo[**‖] and Luigi Vitagliano[‡]

[†]Institute of Molecular Biology and Pathology, CNR c/o Dep. Chemistry, University of Rome, Sapienza, P.le A. Moro 5, 00185 Rome, Italy.
[‡]Institute of Biostructures and Bioimaging, CNR, Via Pietro Castellino 111, 80131 Naples, Italy.
[§]Department of Science and Technology, University of Sannio, via Francesco de Sanctis snc, Benevento 82100, Italy
[‖]Department of Chemistry, University of Rome Sapienza, P.le A.Moro 5, 00185 Rome, Italy.

[*] email:Email: antonella.paladino@cnr.it.
[**] email:Email: marco.dabramo@uniroma1.it.

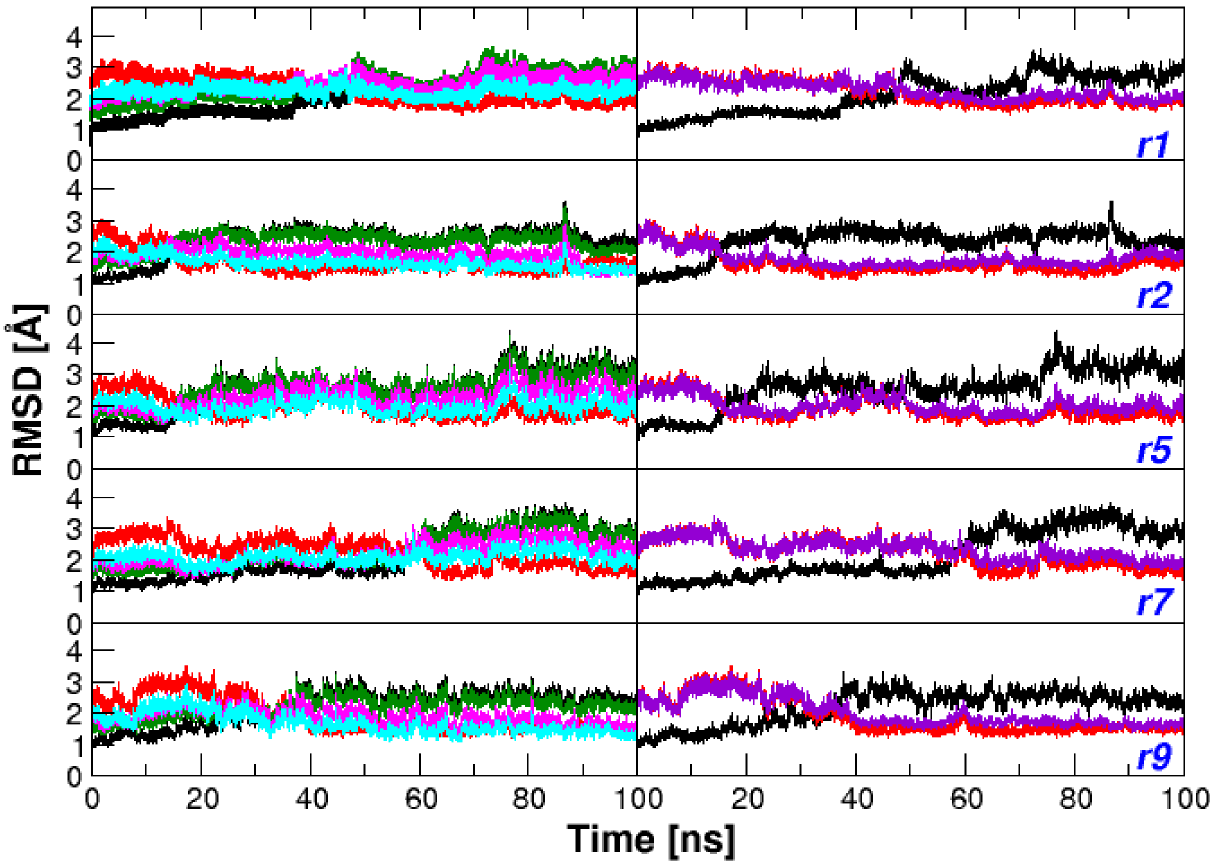

Figure S1. Root mean square deviation (RMSD) analysis of the HbTb simulation runs with observed T→ R transition. RMSD values (on C^{α} atoms) computed against the intermediate states TnA (dark green, PDB ID: 5LFG), TnB (magenta, PDB ID: 5LFG), and TnH (cyan, PDB ID: 3D1K) of Hb from *T. Newnesi* (left panel) and against HL-(C) (violet, PDB ID: 4N7P) of HbA (right panel). For comparison, RMSD values calculated *versus* the starting T model (black, PDB ID: 2H8F) and the R-state (red, PDB ID: 1PBX) of HbTb are also reported.

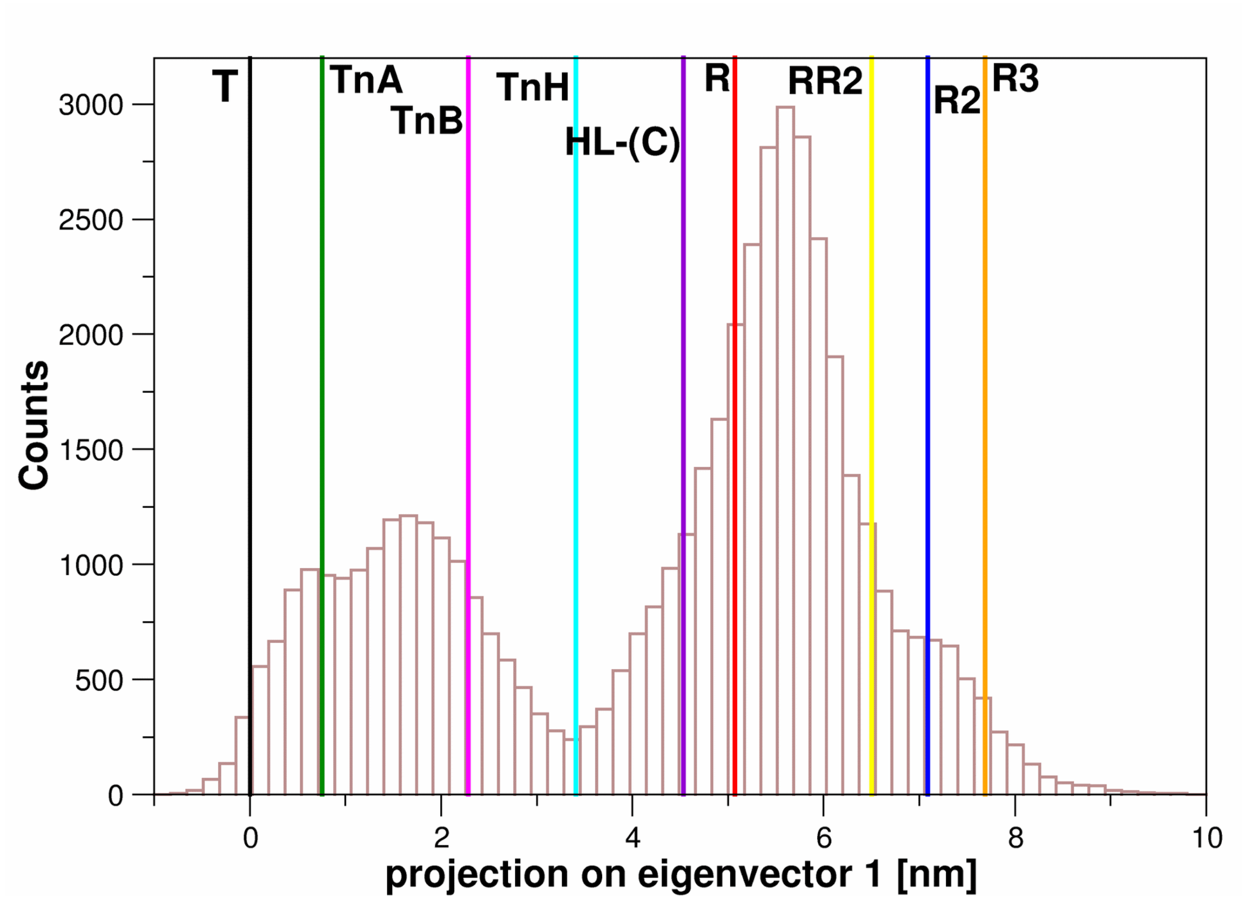

Figure S2. Essential dynamics analysis. Projection on the first eigenvector of the trajectory obtained by concatenating MD runs with observed T→R transition, namely r1, r2, r5, r7 and r9.

The vertical solid lines correspond to the projections of the crystallographic structures of HbTb states: T (black, PDB ID: 2H8F), R (red, PDB ID: 1PBX), HbTn intermediates: TnA (dark green, PDB ID: 5LFG), TnB (magenta, PDB ID: 5LFG), and TnH (cyan, PDB ID: 3D1K), HbA states: intermediate HL-(C) (violet, PDB ID: 4N7P), R2 (blue, PDB ID: 1BBB), RR2 (yellow, PDB ID: 1MKO), and R3 (orange, PDB ID: 4NI0).

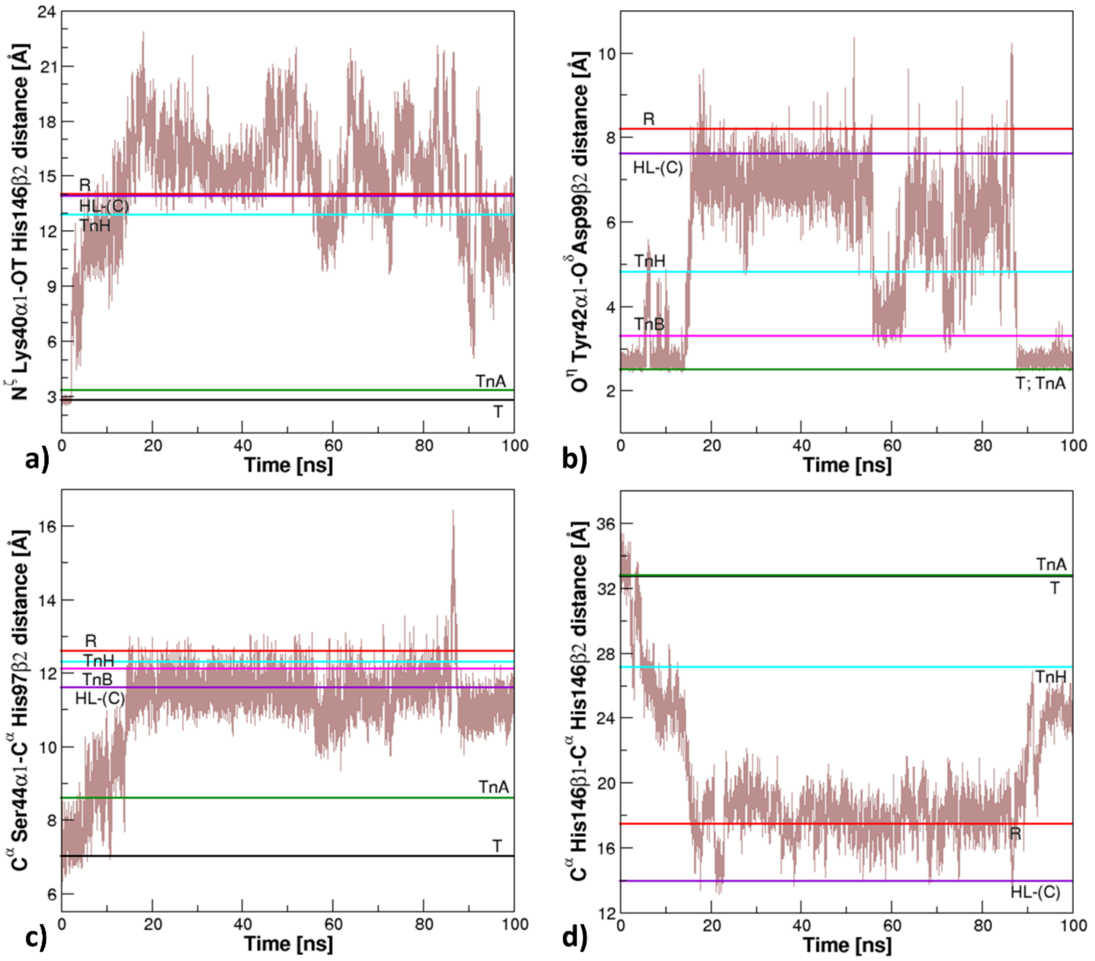

Figure S3. Time evolution of the structural probes that are characteristic of the different HbTb states in r2 simulation run. Specifically, the distances (a) N^{ζ} Lys40α1−OT His146β2, (b) O^{η} Tyr42α1−O^{δ} Asp99β2, (c) C^{α} Ser44α1−C^{α} His97β2, and (d) C^{α} His146β1−C^{α} His146β2 are monitored.

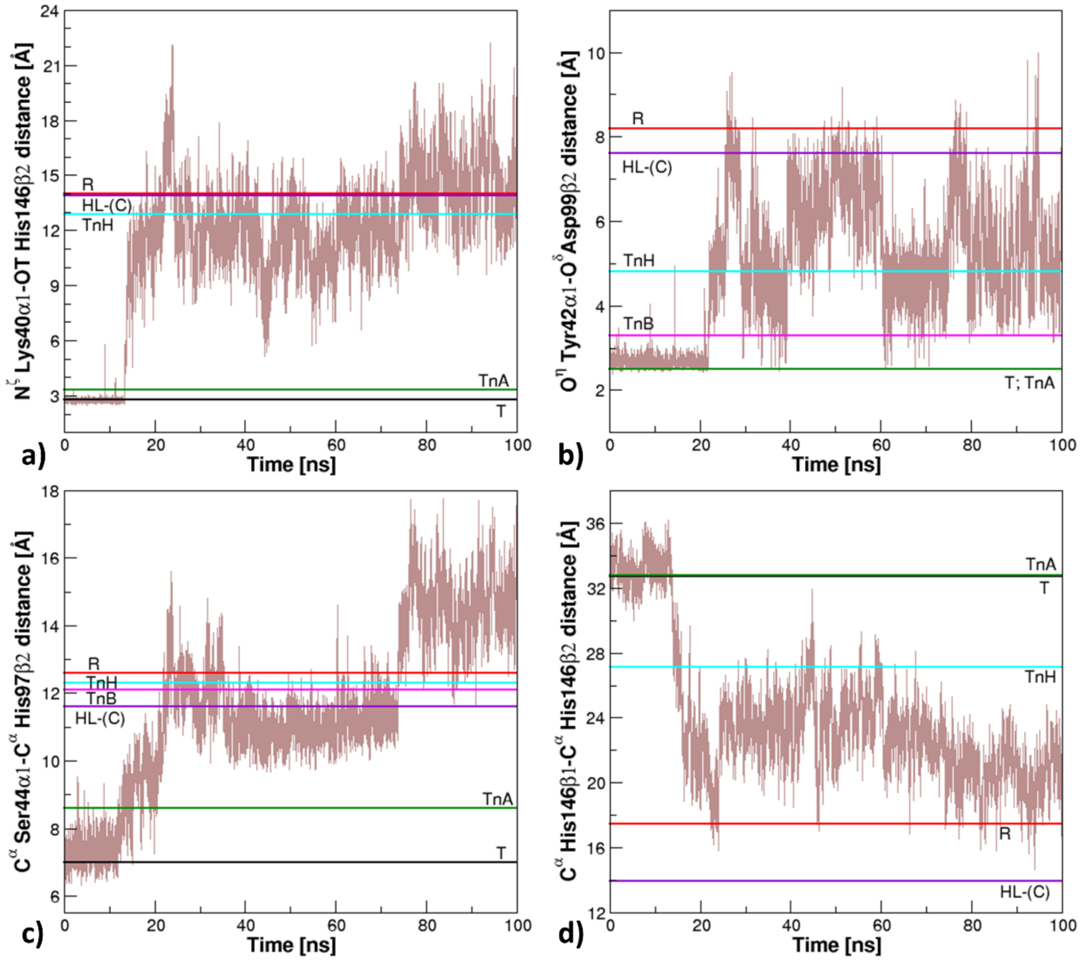

Figure S4. Time evolution of the structural probes that are characteristic of the different HbTb states in r5 simulation run. Specifically, the distances (a) N$^{\zeta}$ Lys40α1−OT His146β2, (b) O$^{\eta}$ Tyr42α1−O$^{\delta}$ Asp99β2, (c) C$^{\alpha}$ Ser44α1−C$^{\alpha}$ His97β2, and (d) C$^{\alpha}$ His146β1−C$^{\alpha}$ His146β2 are monitored.

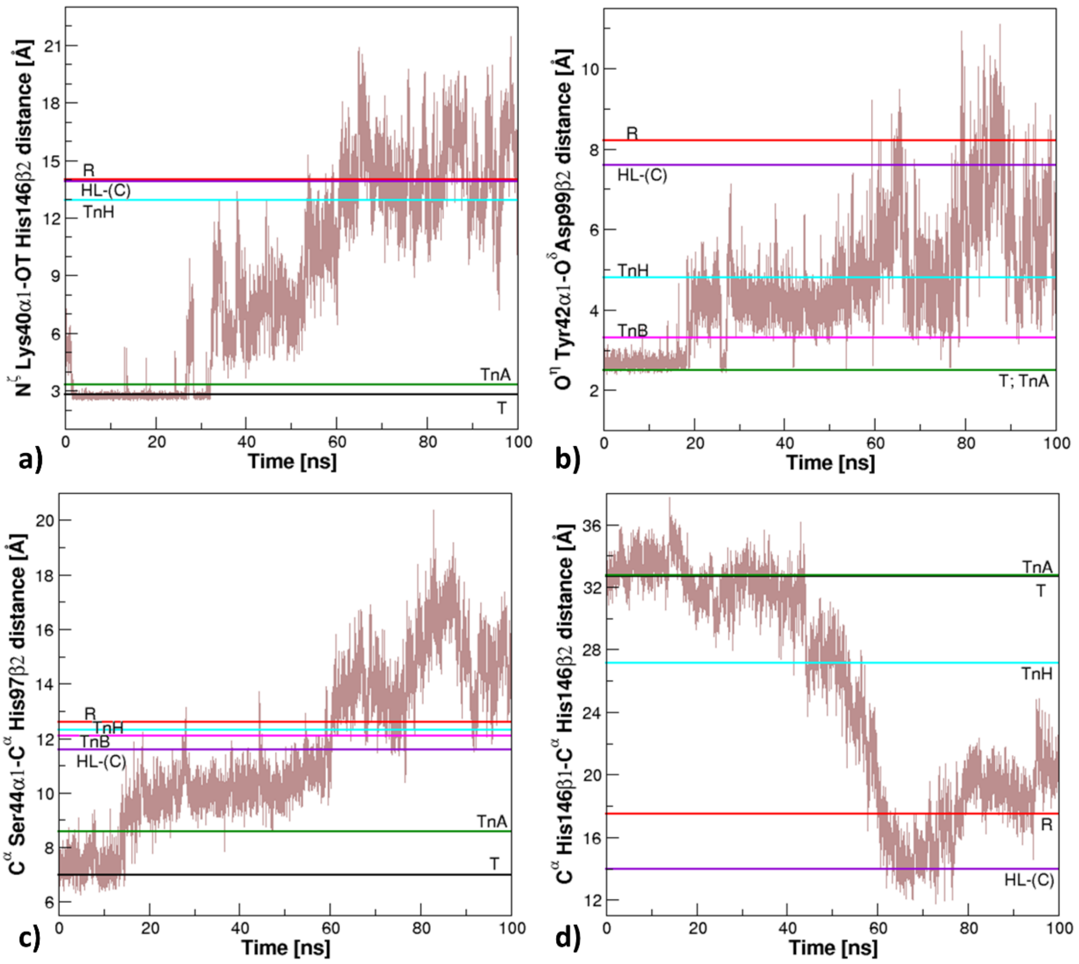

Figure S5. Time evolution of the structural probes that are characteristic of the different HbTb states in r7 simulation run. Specifically, the distances (a) N$^{\zeta}$ Lys40α1−OT His146β2, (b) O$^{\eta}$ Tyr42α1−O$^{\delta}$ Asp99β2, (c) C$^{\alpha}$ Ser44α1−C$^{\alpha}$ His97β2, and (d) C$^{\alpha}$ His146β1−C$^{\alpha}$ His146β2 are monitored.

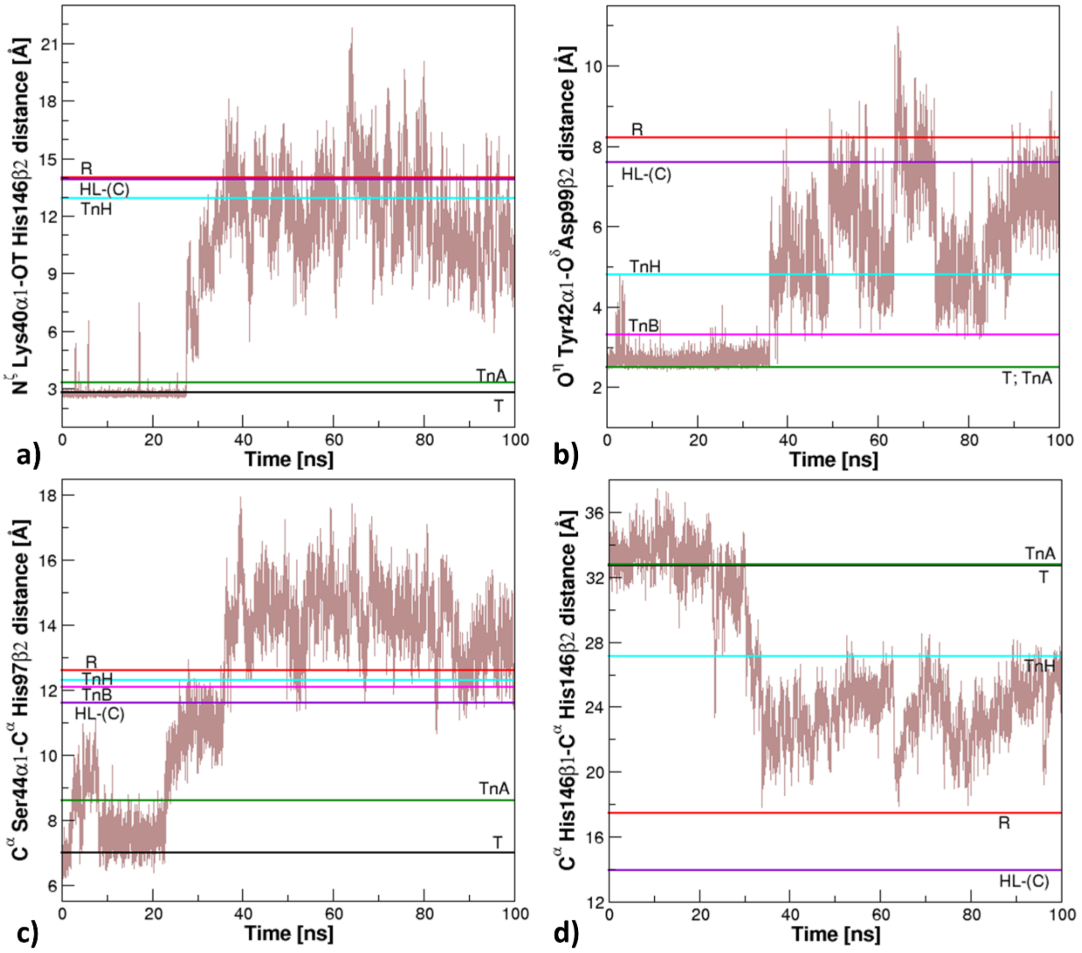

Figure S6. Time evolution of the structural probes that are characteristic of the different HbTb states in r9 simulation run. Specifically, the distances (a) N$^{\zeta}$ Lys40α1−OT His146β2, (b) O$^{\eta}$ Tyr42α1−O$^{\delta}$ Asp99β2, (c) C$^{\alpha}$ Ser44α1−C$^{\alpha}$ His97β2, and (d) C$^{\alpha}$ His146β1−C$^{\alpha}$ His146β2 are monitored.

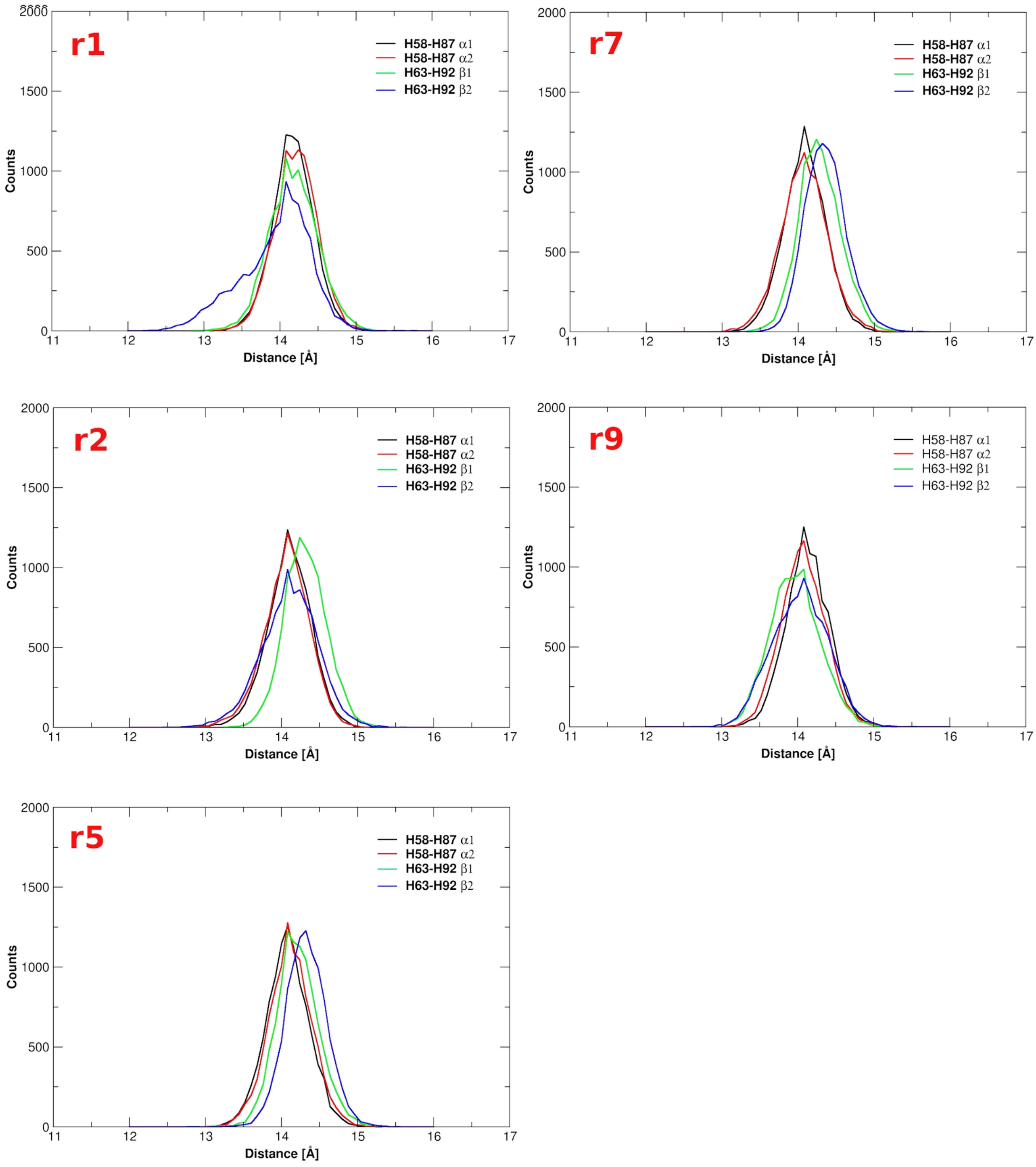

Figure S7. Distributions of the C^{α} -C^{α} distance between the distal and proximal His residues in α1, α2 (His58-His87) and β1, β2 (His63-His92) chains in the simulation runs with observed T→ R transition.

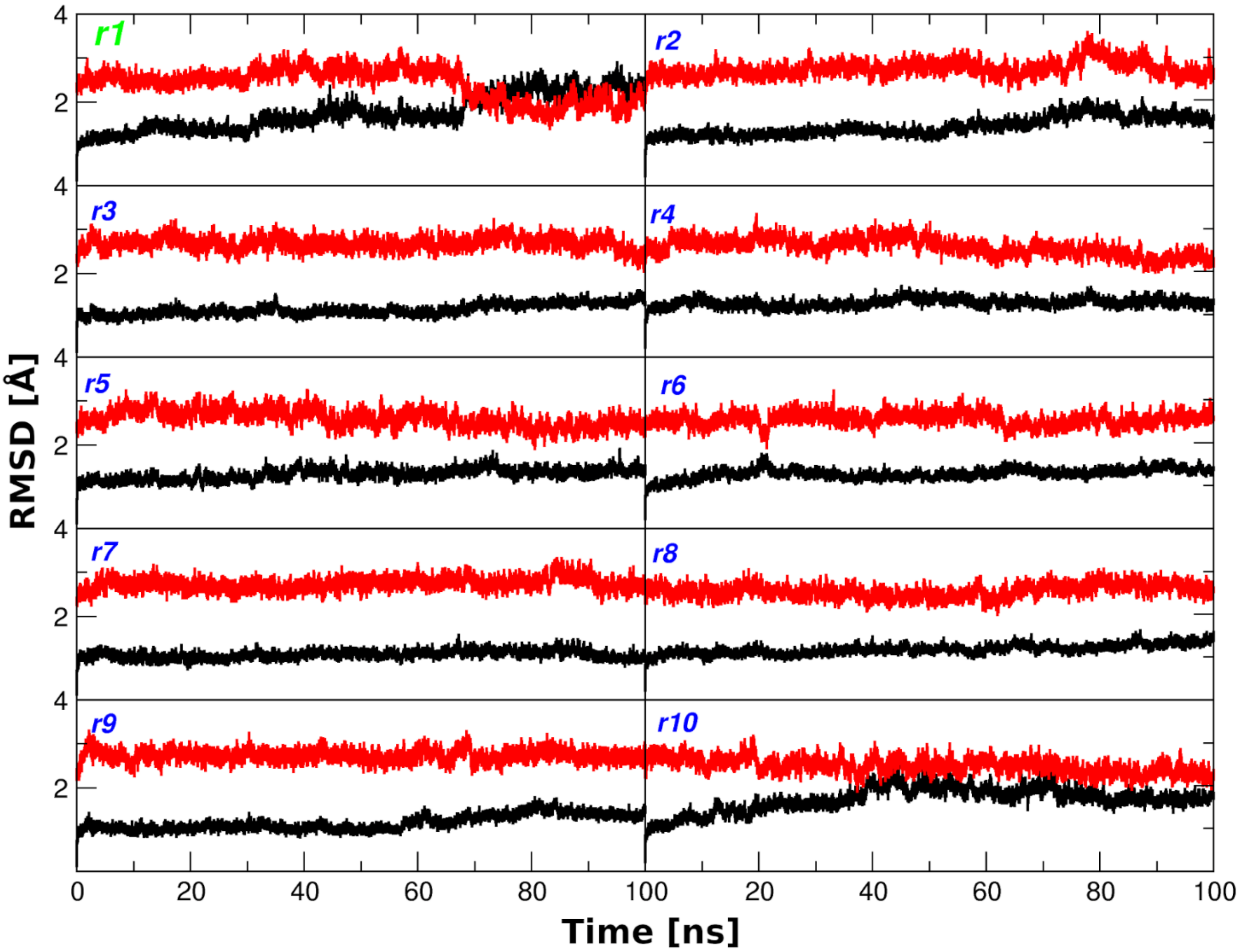

Figure S8. Root mean square deviation (RMSD) analysis of the HbTb simulations carried at T= 273 K. RMSD values (computed on C^{α} atoms) of the trajectory structures *versus* the starting T model (black, PDB ID: 2H8F) and the R-state (red, PDB ID: 1PBX). The simulation run showing the T→ R transition is labeled in green.

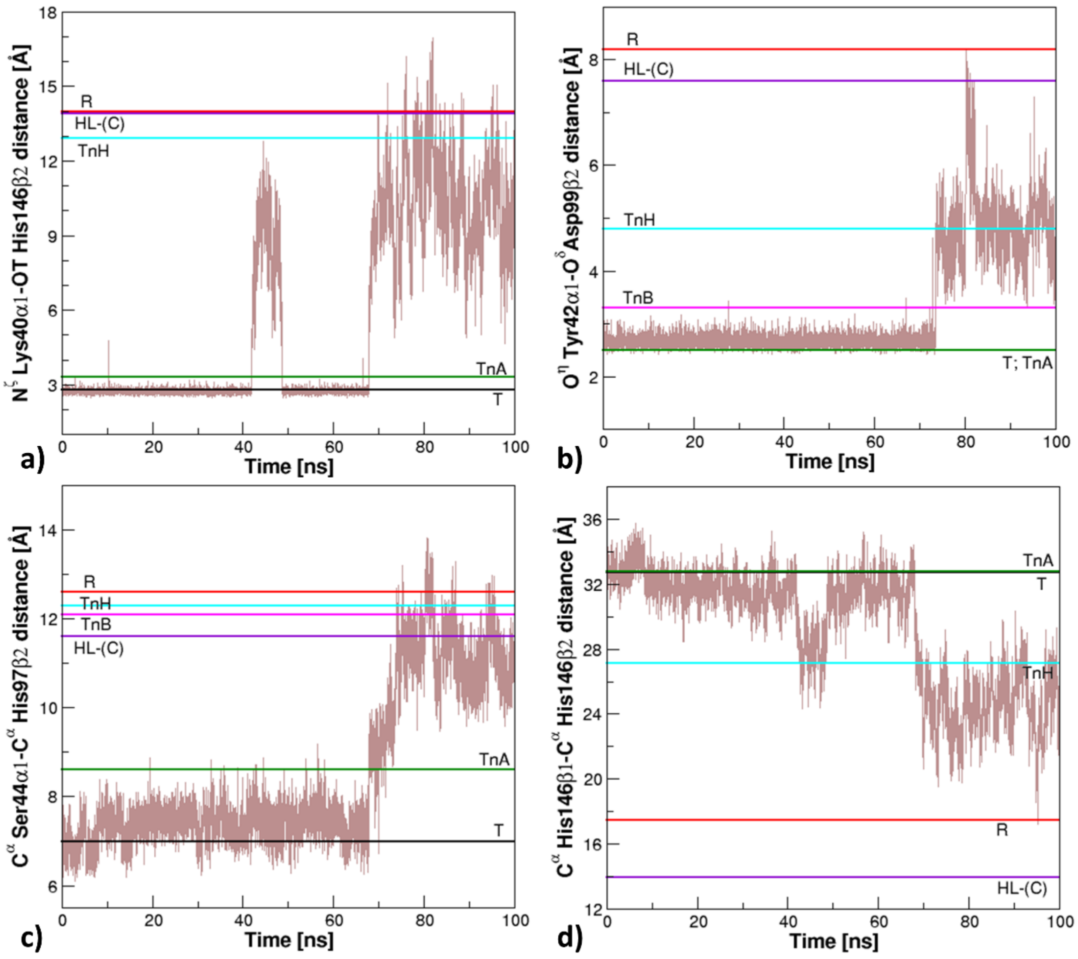

Figure S9. Time evolution of the structural probes that are characteristic of the different HbTb states in $r1_L$ simulation run at T= 273 K. Specifically, the distances (a) N^{ζ} Lys40α1−OT His146β2, (b) O^{η} Tyr42α1−O^{δ} Asp99β2, (c) C^{α} Ser44α1−C^{α} His97β2, and (d) C^{α} His146β1−C^{α} His146β2 are monitored.

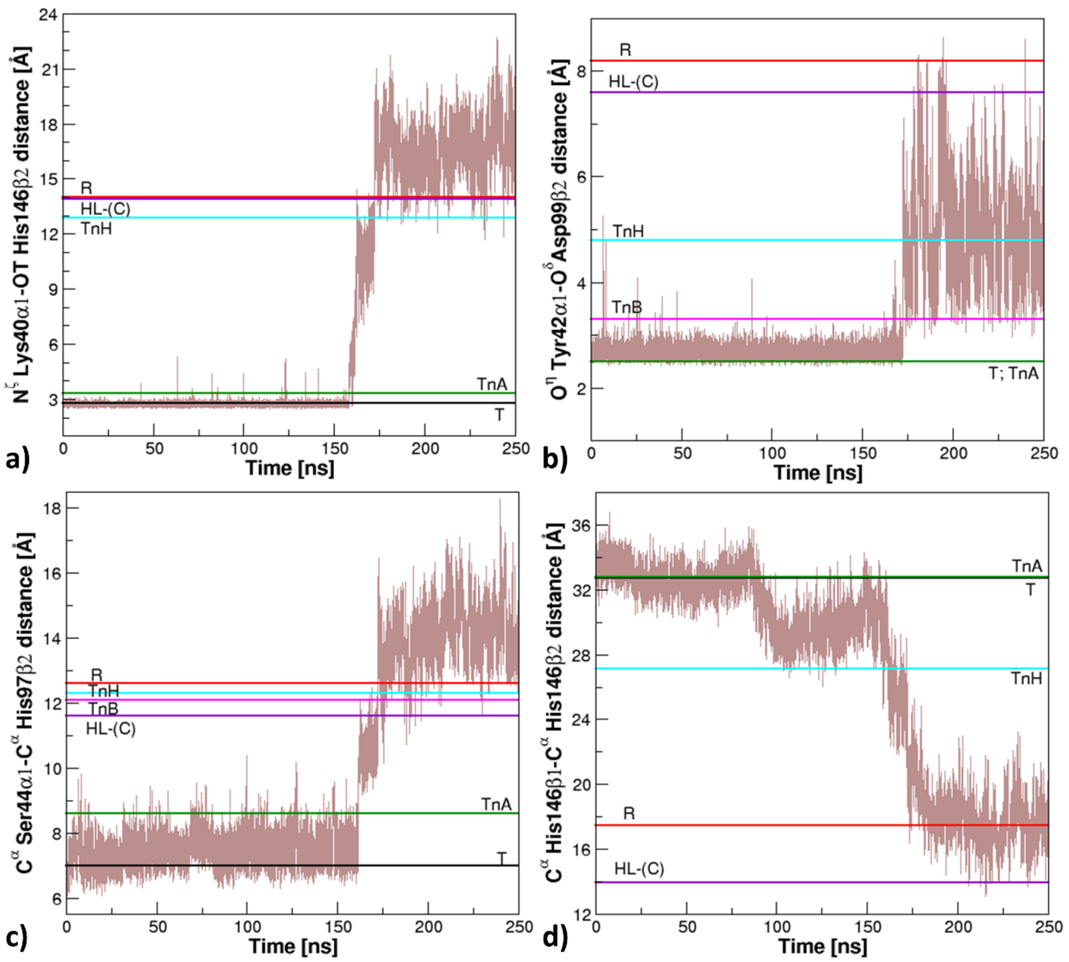

Figure S10. Time evolution of the structural probes that are characteristic of the different HbTb states in r1$_L$* simulation run at T= 273 K. Specifically, the distances (a) N$^{\zeta}$ Lys40α1−OT His146β2, (b) O$^{\eta}$ Tyr42α1−O$^{\delta}$ Asp99β2, (c) C$^{\alpha}$ Ser44α1−C$^{\alpha}$ His97β2, and (d) C$^{\alpha}$ His146β1−C$^{\alpha}$ His146β2 are monitored.

Table S1. Details of representative crystallographic structures of HbTb (T and R), HbA (R2, RR2, R3 and HL-(C)) and HbTn (TnA, TnB and TnH).

**Hb State**	**PDB code**	**Resol. (Å)**	**R/Rfree**	**Asymmetric unit**	**Binding state**
T	2H8F	1.30	0.151/0.172	tetramer	Unliganded
R	1PBX	2.50	0.178/null	dimer	Fully-liganded (CO)
R2	1BBB	1.7	0.184/null	tetramer	Fully-liganded (CO)
RR2	1MKO	2.18	0.200/0.284	tetramer	Fully-liganded (CO)
R3	4NI0	2.15	0.228/0.259	dimer	Fully-liganded (CO)
HL-(C)	4N7P	2.8	0.259/0.287	3 tetramers: HL-(A), HL-(B), HL-(C)	Half-liganded (CO)
TnA, TnB	5LFG	1.94	0.181/0.245	2 dimers: TnA, TnB	Fully-liganded (CO)
TnH	3D1K	1.25	0.167/0.202	dimer	Half-liganded (CO)
